# Functional Characterization of the Small Heat Shock Protein Hsp12p from *Candida albicans*

**DOI:** 10.1371/journal.pone.0042894

**Published:** 2012-08-07

**Authors:** Man-Shun Fu, Luisa De Sordi, Fritz A. Mühlschlegel

**Affiliations:** 1 School of Biosciences, University of Kent, Canterbury, Kent, United Kingdom; 2 Clinical Microbiology Service, East Kent Hospitals University NHS Foundation Trust, Ashford, Kent, United Kingdom; Yonsei University, Republic of Korea

## Abstract

Hsp12p is considered to be a small heat shock protein and conserved among fungal species. To investigate the expression of this heat shock protein in the fungal pathogen *Candida albicans* we developed an anti-*Ca*Hsp12p antibody. We show that this protein is induced during stationary phase growth and under stress conditions including heat shock, osmotic, oxidative and heavy metal stress. Furthermore, we find that *Ca*Hsp12p expression is influenced by the quorum sensing molecule farnesol, the change of CO_2_ concentration and pH. Notably we show that the key transcription factor Efg1p acts as a positive regulator of *Ca*Hsp12p in response to heat shock and oxidative stress and demonstrate that *Ca*Hsp12p expression is additionally modulated by Hog1p and the cAMP-PKA signaling pathway. To study the function of Hsp12p in *C. albicans* we generated a null mutant, in which all four Ca*HSP12* genes have been deleted. Phenotypic analysis of the strain shows that Ca*HSP12* is not essential for stress resistance, morphogenesis or virulence when tested in a *Drosophila* model of infection. However, when overexpressed, Ca*HSP12* significantly enhanced cell-cell adhesion, germ tube formation and susceptibility to azole antifungal agents whilst desensitizing *C. albicans* to the quorum sensing molecule farnesol.

## Introduction

*Candida albicans* is an ascomycete yeast which can be found in the gastrointestinal tract and the oral or vaginal mucosa of many otherwise healthy individuals [1]. However, it is also a major opportunistic fungal pathogen, causing superficial infections of mucosa and skin, or life-threatening invasive infections when either the innate or acquired immune system is compromised [1], [2].

Prompt sensing and adaptation to a wide range of environmental conditions are key for fungal survival in the various niches of the host and *C. albicans* has developed a fine-tuned response to stresses required for efficient host colonization [3], [4], [5], [6], [7]. The ability to undergo a reversible morphological transition in response to environmental changes is an additional advantage of *C. albicans* host adaptation. In fact, both stress response and polymorphism are considered major virulence factors of *C. albicans* [8], [9]. Previous studies have shown that the Hog1p MAPK signaling pathway plays an important role in regulation of stress response [8] whereas *C. albicans* polymorphism is controlled by multiple signaling pathways including the Efg1p-mediated cAMP pathway, Cph1p-mediated MAPK, Rim101p and Tup1p dependant pathways [6], [10], [11], [12].

Heat shock proteins, a group of molecular chaperones found in all organisms, accumulate upon a shift from physiological to higher temperatures. They are also induced by other stresses and thus allow the cells to survive under challenging conditions [13]. Heat shock proteins are classified based on their molecular weight [14]. Small heat shock proteins have a molecular mass ranging between 10 and 30 kDa and share a conserved sequence in their C-terminus called the α-cystallin domain [15], [16]. Yeast small heat shock proteins, including Hsp26p and Hsp30p, are induced under heat shock and during stationary growth phase [17], [18]. Over-expression of Hsp26p increases thermo-tolerance of yeast cells [19] and *C. albicans* Hsp30p has been shown to be induced upon exposure to the antifungal agent amphotericin B [20].

*C. albicans HSP12* (Ca*HSP12*) gene expresssion is regulated by changes in the concentrations of environmental CO_2_ and pH via the cAMP-dependent and Rim101p-dependent signaling cascades [21]. Other reports have shown that Ca*HSP12* is induced when cells are exposed to osmotic stress, oxidative stress, heavy metal stress and heat shock [8], [22], [23]. Additionally, Ca*HSP12* expression is regulated by quorum sensing molecules [24], upon hypoxic conditions [25], drug-resistance [26], [27], tissue invasion [28], the yeast-to-hyphal transition [29] and iron limitation [30]. *HSP12* in *Candida glabrata*, which is the second most common cause of systemic candidiasis, is up-regulated in fluconazole-resistant mutants [31]. Finally, *HSP12* orthologs in *Cryptococcus neoformans* which is another pathogenic fungus have role in polyene antifungal drug susceptibility and are regulated by the cAMP signaling pathway [32].

Despite this large amount of information gathered on *HSP12* gene expression very little is known about its function in fungal species in general and nothing on the *C. albicans* Hsp12p protein in particular. In this study, we characterize Ca*HSP12* from the fungal pathogen *C. albicans* with respect to its gene structure, regulation of protein expression, function and virulence. We show that *Ca*Hsp12p is induced by stress and the quorum sensing molecule farnesol, and regulated by the change of CO_2_ concentration and pH. Notably, we identify the transcription factor Efg1p to be required for expression in response to heat shock and oxidative stress and demonstrate that expression of *Ca*Hsp12p is additionally regulated by the Hog1p and cAMP signaling pathways. We also present a comparative study on *HSP12* expression in *C. albicans*, *S. cerevisiae* and *C. glabrata* in response to different stresses in general, and report differences among these yeast species when exposed to oxidative stress in particular. We find that Hsp12p is not essential for stress resistance, filamentation or virulence. However, when overexpressed, it enhances cell-cell aggregation, susceptibility to azole antifungal agents, and promotes farnesol tolerance.

## Materials and Methods

### Strains and growth conditions

The yeast strains used in this study are listed in Table 1. All strains were grown either in rich YEPD medium or in YNB minimal medium or YNB minimal medium buffered with 150 mM HEPES as described [5], [21]. All *C. albicans* and *C. glabrata* strains were grown at 37°C unless indicated otherwise. All *S. cerevisiae* strains were grown at 30°C.

**Table 1:** Yeast strains used in this study.

Strain	Description	Genotype	Source
SC5314	*C. albicans* laboratory wild-type strain		
CAI4	*URA3* auxotrophic strain	*ura3*:: *λimm434/ura3*:: *λimm434*	[33]
BWP17	*URA3*, *HIS1*, *ARG4* auxotrophic strain	*ura3:: λimm434/ura3*:: *λimm434 his1*::*hisG/his1*::*hisG arg4*::*hisG/arg4*::*hisG*	[34]
CAI4-pFM2	Wild-type strain transformed with pFM2 as the control in *HSP12* overexpression experiment	*ura3*:: *λimm434/ura3*:: *λimm434*-(pFM2 *URA3*)	This study
BWT	With-type strain transformed with CIp30 as the control in *CaHSP12* deletion experiment	*ura3:: λimm434/ura3*:: *λimm434 his1*::*hisG/his1*::*hisG arg4*::*hisG/arg4*::*hisG rps1*-(CIp30 *URA3, HIS1, ARG4*)	This study
HSP12OE	*CaHSP12* overexpressing strain	*ura3*:: *λimm434/ura3*:: *λimm434*-(*CaHSP12*-pFM2 *CaHSP12*, *URA3*)	This study
HSP12KO2	Strain with two *CaHSP12* alleles deleted	*ura3:: λimm434/ura3*:: *λimm434 his1*::*hisG/his1*::*hisG arg4*::*hisG/arg4*::*hisG hsp12a::HIS1/hsp12a::hisG-URA3-hisG, HSP12b/HSP12b*	This study
HSP12KO5	*CaHSP12* deletion strain transformed with CIp30	*ura3:: λimm434/ura3*:: *λimm434 his1*::*hisG/his1*::*hisG arg4*::*hisG/arg4*::*hisG hsp12a::HIS1/hsp12a::hisG, hsp12b::hisG/hsp12b::hisG rps1*-(CIp30 *URA3, HIS1, ARG4*)	This study
HSP12C	*CaHSP12* reconstitution strain	*ura3:: λimm434/ura3*:: *λimm434 his1*::*hisG/his1*::*hisG arg4*::*hisG/arg4*::*hisG hsp12a::HIS1/hsp12a::hisG, hsp12b::hisG/hsp12b::hisG rps1*-(*CaHSP12-*CIp30 *CaHSP12, URA3, HIS1, ARG4*)	This study
Cg2001	*C. glabrata* wild-type		
Cg2001TU	*C. glabrata TRP1 URA3* auxotrophic strain	*Δura3 Δtrp1*	[38]
Cg12KO	*CgHSP12* deletion strain	*Δura3 Δtrp1 ΔCghsp12::TRP1* (pEM13D *URA3*)	This study
Cg12C	*CgHSP12* reconstitution strain	*Δura3 Δtrp1 ΔCghsp12::TRP1 –(CgHSP12*-pEM13D *CgHSP12*, *URA3*)	This study
BY4741	*S. cerevisiae HIS3 LEU2 MET15 URA3* auxotrophic strain	*MAT*a *his3*Δ*1 leu2*Δ*0 met15*Δ*0 ura3*Δ*0*	[63]

### Strain construction

For a comprehensive description of all methods see Text S1. Briefly, both *HSP12* loci (designated Ca*HSP12a* and Ca*HSP12b*) present in the *C. albicans* genome were deleted by using Ura-blaster and *HIS1* cassettes in BWP17 strain according to standard protocols [33], [34], [35]. Reconstitution strains (HSP12C) were constructed by integrating CIp30 containing a wild-type copy of Ca*HSP12* to the *RP10* locus [36]. To construct the *CaHSP12* overexpressing strain, HSP12OE, Ca*HSP12* was cloned downstream of the *TEF2* promoter in pFM2 [3]. *C. glabrata HSP12* was cloned, disrupted and reconstituted according to standard protocols [37], [38].

### Anti-*Ca*Hsp12p antibody generation

*Ca*Hsp12p was expressed in *E. coli* and purified using GST-tag affinity chromatography. Purified *Ca*Hsp12p was then boiled at 95°C for 10 min before sending to Charles River Laboratories (Romans-sur-Isère, France). Antibody generation, protein expression in yeast and Western blotting are detailed in Text S1 and as previously described [5].

### Phenotypic assays

Growth rate determination, cell-cell aggregation and adhesion studies using the XTT reduction assays, farnesol susceptibility studies, antifungal drug and stress sensitivity tests and virulence test using our previously published *Drosophila* model [7] are all describe in Text S1.

## Results

### *C. albicans* contains two Ca*HSP12* genes

**Figure 1:**
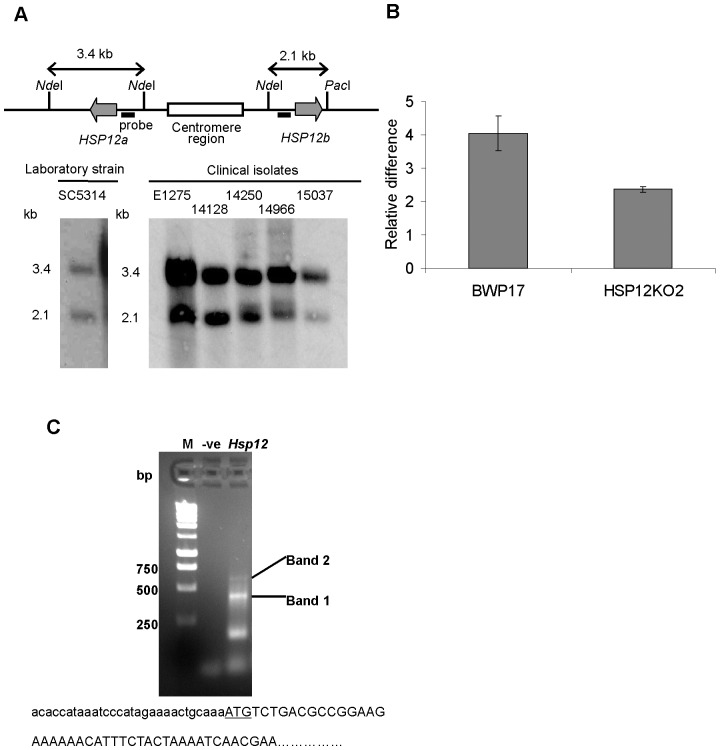
*HSP12* differs among yeast species. (A) Two Ca*HSP12* genes have been identified in *C. albicans* SC5314 and five clinical isolates by Southern blot. (B) Both alleles of Ca*HSP12* are transcriptionally expressed. The transcription level of Ca*HSP12* is assessed by qRT-PCR of total RNA obtained from the strain with absence of one Ca*HSP12* gene (HSP12KO2) and its parental strain (BWP17). The error bars represent the S.D. of triplicate independent reactions. *P* value<0.01, two-sided unpaired student t-test. (**C**) 5′ RACE analysis of Ca*HSP12*. Two DNA bands (band 1 and band 2) with the expected size above 250 bp were sequenced. The sequencing shows that the 5′ untranslated region (in lowercase) contains 29 bp nucleotides and only the second start codon (underlined) of *CaHSP12* can be identified. M: 1 kb DNA ladder; -ve: negative control of PCR without template; *HSP12*: 5′RLM-RACE PCR product of *CaHSP12*.

We identified two loci of Ca*HSP12*, arranged in an inverted manner (Ca*HSP12a* and Ca*HSP12b* with GenBank Accession Nos. XM715434 and XM709485), in the *C. albicans* genome database. Both genes are located within 6.5 kb of each other in proximity to the centromere on chromosome 5. Their predicted open reading frames encode proteins that differ in only two amino acids. The homology of the upstream regions (1640 bp) of the two different loci is 99%. However, the 1000 bp downstream regions are only <50% identical. Due to this dissimilarity, different sizes of *Nde*I-*Pac*I digested fragments of the two Ca*HSP12* copies (3.4 kb and 2.1 kb) are obtained and visualized in Southern blots (Figure 1A). We confirmed that not only the *C. albicans* type strain, SC5314, but equally five additional clinical isolates carry two loci of different Ca*HSP12* (Figure 1A). Database sequences show that there are two *HSP12* genes present in the genome of *Candida dublinensis*, which is closely related to *C. albicans*, but only one in *S. cerevisiae*, *C. glabrata*, *Candida tropicalis*, *Candida guiliermondii*, *Candida lusitaniae* and *Cryptococcus neoformans*. To determine if both copies of Ca*HSP12* are expressed, qRT-PCR was carried out in a strain in which one copy of Ca*HSP12* had been deleted (HSP12KO2). This showed a reduction of Ca*HSP12* expression by 50% compared to the parent strain (Figure 1B) suggesting that both copies are expressed in *C. albicans*.

### *C. albicans HSP12* contains two putative start codons

**Figure 2:**
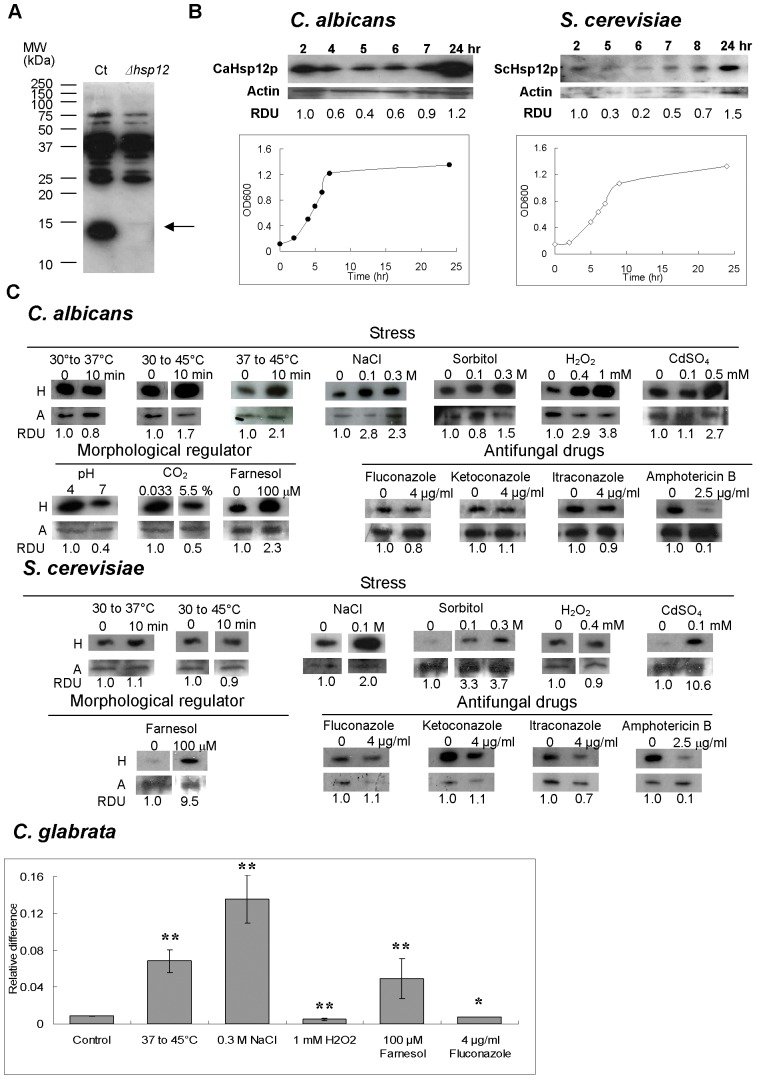
Hsp12p is regulated in response to a wide range of stresses. (A) *Ca*Hsp12p is recognized by a polyclonal antibody. The anti-*Ca*Hsp12p antibody was tested by using Western blot analysis against protein samples from the *C. albicans* CAI4 control strain (Ct) and the *Cahsp12* null mutant (*Δhsp12*). The arrow indicates the 13 kDa band of *Ca*Hsp12p which is present in CAI4, but absent in the *Cahsp12* null mutant. (B) Induction of Hsp12p in *C. albicans* and in *S. cerevisiae* during stationary growth. Total protein was extracted at the indicated time points from *C. albicans* CAI4 at 37°C or *S. cerevisiae* BY4741 at 30°C. Western blots were probed with anti-Hsp12p antibody and showed a band corresponding to the expected size of 13 kDa. Blots were probed with anti-actin antibody as loading control. Growth curves with sampling time points (open or solid dots) are shown. RDU: relative densitometry units. (C) Hsp12p is regulated in response to diverse conditions. Hsp12 protein level in *C. albicans* CAI4 or *S. cerevisiae* BY4741 was assayed using Western blot and a band of the expected size (13 kDa) was detected. H: anti-Hsp12p antibody. A: anti-actin antibody (equal protein loading control). RDU: relative densitometry units. *CgHSP12* transcript level was determined by qRT-PCR with total RNA extracted from the Cg2001TU strain. The transcript level was normalized to the *Act1* control. The error bars represent the S.D. of triplicate independent reactions. **P value<0.01, * *P* value>0.3, two-sided unpaired student t-test.

Bioinformatics analysis identified two putative start codons (ATG) for both Ca*HSP12* loci while only one is found in *HSP12* from other fungal species. Translation from the first start codon would produce a 168 amino acid protein corresponding to a 18.0 kDa protein whereas translation from the second would lead to a 127 amino acid, 13 kDa, protein. The origin of transcription was determined by analysis of the 5′ end of Ca*HSP12* mRNA via sequence analysis of 5′ RACE reaction products (Figure 1C). This revealed that the 5′ start point of the Ca*HSP12* transcript is present at position −29 from the second start codon (Figure 1C). Western blot analysis, using an anti-Hsp12p antibody, identified a signal with a size of 13 kDa (Figure 2A).

### *C. albicans* Hsp12p is regulated in response to a wide range of stresses

To study *C. albicans* cells that were exposed to different stresses we raised an anti-*Ca*Hsp12p antibody (Figure 2A). Furthermore, we compared the expression of Hsp12p between *C. albicans* and *S. cerevisiae* using an *S. cerevisiae* anti-Hsp12 antibody. Finally, we studied the expression of Cg*HSP12* in *C. glabrata*, which is phylogenetically closely related to *S. cerevisiae*.

Heat shock proteins in fungi are synthesized at high levels during stationary phase growth [14], [17], [39] and we show that protein expression of *Ca*Hsp12p is highly induced in stationary phase but not in exponentially growth of *C. albicans* (Figure 2B). We also show that *Sc*Hsp12p is increased in stationary phase (Figure 2B), confirming previous northern blot analysis of Sc*HSP12* transcript by Praekelt and Meacock [14].

Transcription of *HSP12* has been shown to be induced under stress in both *S. cerevisiae* and *C. albicans* [8], [14], [22], [23], [40], [41]. Hence, we examined the response of Hsp12p to stress at the protein level. Western blot analysis showed that *Ca*Hsp12p is induced by heat shock (from 30°C to 45°C or from 37°C to 45°C), however no induction of *Ca*Hsp12p was observed when shifting the temperature from 30°C to 37°C (Figure 2C). Apart from heat shock, *Ca*Hsp12p is also induced in osmotic stress such as sodium chloride (NaCl) and sorbitol, oxidative stress such as hydrogen peroxide (H_2_O_2_) , and the heavy metal cadmium (Cd^2+^) (Figure 2C). We noted that even exposure to low doses of NaCl (0.1 M) or H_2_O_2_ (0.4 mM) resulted in significant induction of *Ca*Hsp12p expression (Figure 2C). However, *Ca*Hsp12p is slightly increased when grown in high concentrations of sorbitol (0.3 M) (Figure 2C). Expression is only enhanced in cells treated with higher levels of heavy metal Cd^2+^ (0.5 mM), but not in the lower doses (0.1 mM Cd^2+^) (Figure 2C). Similar to *Ca*Hsp12p, *Sc*Hsp12p is induced by osmotic stress such as low doses of NaCl (0.1 M) and sorbitol (0.1 M), and heavy metal Cd^2+^ (0.1 mM) (Figure 2C). However, we found that *Sc*Hsp12p is not induced when shifting cells from 30°C to either 37°C or 45°C (Figure 2C). Interestingly, unlike *Ca*Hsp12p, *Sc*Hsp12p was not regulated by H_2_O_2_ (Figure 2C). Analysis of *C. glabrata HSP12* transcript levels revealed an 8-fold induction following heat shock from 37°C to 45°C (Figure 2C). Cg*HSP12* was 15-fold up-regulated after exposure to 0.3 M NaCl (Figure 2C) but the transcript level was slightly decreased (2-fold) following exposure to 1 mM H_2_O_2_ (Figure 2C). Finally we show that *C. albicans* Hsp12 protein is down-regulated by physiological CO_2_ and pH (Figure 2C).

### *C. albicans, S. cerevisiae and C. glabrata* Hsp12p is induced by the quorum sensing molecule farnesol

Using qRT-PCR, Davis-Hanna *et al.* have previously shown that transcription of Ca*HSP12* was influenced when *C. albicans* was grown in the presence of the quorum sensing molecule farnesol [24]. Consistent with this work we show that *Ca*Hsp12p protein levels sharply increase in response to 100 µM farnesol (Figure 2C). Intriguingly, *Sc*Hsp12p is also highly induced (Figure 2C), and Cg*HSP12* is 6-fold increased upon exposure to farnesol (Figure 2C).

### Polyene but not azole antifungal agents impact on *Ca*Hsp12p expression

Previous work by Coste *et al* has shown that the promoter of Ca*HSP12* contains a *cis*-acting drug-responsive element (DRE)-like region with four mismatches [26]. Additionally, Ca*HSP12* was found to be up-regulated in azole-resistant strains [27], [42]. Moreover, Ca*HSP12* is induced when the cells are exposed to fluphenazine, which can also induce multidrug transporter genes [27]. However, there is no direct evidence showing if Ca*HSP12* is regulated by antifungal drugs. Therefore, we investigated whether *Ca*Hsp12p is regulated when the cells were treated with 4 µg ml^−1^ of the azole drugs fluconazole, ketoconazole, itraconazole, or 2.5 µg ml^−1^ of the polyene antifungal agent amphotericin B. No significant change of *Ca*Hsp12p level was been found when *C. albicans* was treated with azole antifungal drugs (Figure 2C). Interestingly, *Ca*Hsp12p is down-regulated upon exposure to amphotericin B (Figure 2C). *C. glabrata HSP12* is also not regulated when the cells were exposed to 4 µg ml^−1^ fluconazole whereas *S. cerevisiae* Hsp12p is slightly down-regulated in the presence of itraconazole and significantly decreased upon exposure to amphotericin B (Figure 2C).

### Hsp12p expression is regulated by the Hog1p stress response and cAMP-PKA signaling pathway

**Figure 3:**
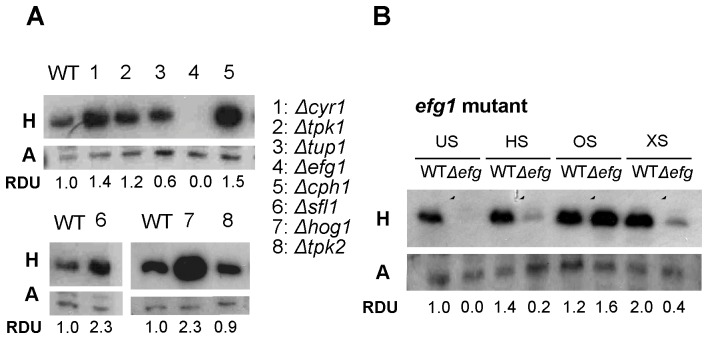
Expression of *Ca*Hsp12p in *C. albicans* mutant strains. (A) *Ca*Hsp12p expression is regulated by the Hog1p stress response and cAMP-PKA signalling pathway. *Ca*Hsp12p was isolated from mutant strains after growing until mid-log phase and its level analyzed by Western blot. Equal protein loading was assessed by probing the blot with anti-actin antibody. H: anti-*Ca*Hsp12p; A: anti-actin; RDU: relative densitometry units. (B) *EFG1* is required for the induction of *Ca*Hsp12p in response to heat shock and oxidative stress. Western blot show the level of *Ca*Hsp12p in the *efg1* mutant in unstressed condition (US) or following exposure to heat shock from 37°C to 45°C (HS), osmotic stress, 0.3 M NaCl, (OS) or oxidative stress, 1 mM H_2_O_2_, (XS). Equal protein loading was assessed by probing the blot with anti-actin antibody. H: anti-*Ca*Hsp12p; A: anti-actin; RDU: relative densitometry units.

The mechanisms of *Ca*Hsp12p regulation during stress response and yeast-to-hyphae transition in *C. albicans* are unclear. Therefore, we determined if *Ca*Hsp12p expression is influenced by protein kinases or key transcription factors which are involved in stress response and regulating filamentation. To this end we monitored expression in the *hog1* [8], *cyr1* [7], *tpk1* [43], *tpk2* [43], *efg1* [44], *cph1* [44], *tup1* [45], and *sfl1* [46] mutants. Notably, expression of *Ca*Hsp12p was repressed in the *efg1* mutant (Figure 3A), suggesting that Efg1p functions as an activator of *Ca*Hsp12p. *Ca*Hsp12p was also slightly repressed in the *tup1* mutant. In contrast, elevated levels of *Ca*Hsp12p were observed in the *hog1*, *cyr1*, *cph1* and, *sfl1*. The level of *Ca*Hsp12p was slightly increased in *tpk1* but not *tpk2* mutants in unstressed conditions, suggesting that Hog1p, Cyr1p, Cph1p, Sfl1p and Tpk1p but not Tpk2p, repress the production of *Ca*Hsp12p (Figure 3A).

### Efg1p is required for the expression of *Ca*Hsp12p during heat shock and oxidative but not osmotic stress

In order to determine the role of Hog1p and cAMP signaling pathway on the regulation of *Ca*Hsp12p in response to stress, expression of *Ca*Hsp12p was examined in the *hog1*, *cyr1*, *tpk1*, *tpk2* and *efg1* deletion mutants after exposure to heat shock from 37°C to 45°C, 0.3 M NaCl and 1 mM H_2_O_2_. Western blots showed that *Ca*Hsp12p levels are reduced in the *efg1* mutant following heat shock and exposure to NaCl, but not H_2_O_2_. This suggested that Efg1p is required for the expression of *Ca*Hsp12p under heat shock and oxidative stress, but not to osmotic stress (Figure 3B). These result indicated that there are distinct mechanisms for osmotic stress response and for heat and oxidative stress response in *C. albicans*. The level of *Ca*Hsp12p expression remained high in the *hog1*, *cyr1* and *tpk1* deletion mutants exposed to stress (Figure S1). Also, the level of *Ca*Hsp12p was not changed between the control strain and the *tpk2* deletion mutant under stress (Figure S1).

### *HSP12* is not essential for growth, stress resistance or virulence

To gain insight into the function of Hsp12p in both *C. albicans* and *C. glabrata* we constructed *hsp12* null mutants in both species. This required deletion of all four *HSP12* alleles in *C. albicans*, and the single gene in *C. glabrata*. Determination of the growth rates or cell adhesion of the Ca*hsp12* and the Cg*hsp12* deletion mutants did not reveal any differences when compared with their control strains (Figure S2). Furthermore, similar growth on medium supplemented with either osmotic stressors such as sodium chloride, sorbitol; oxidative stressors such as H_2_O_2_, menadione; cell wall and cell membrane stressors such as Congo red, calcofluor white, caffeine and SDS or antifungal drugs such as itraconazole, ketoconazole, fluconazole and amphotericin B did not reveal differences in survival or growth (Figure S3 and S4). The Ca*hsp12* deletion mutant did not show any difference in germ tube formation when compared with its control strain (Figure S5). The results indicate that Hsp12p is not essential for the growth, cell adhesion, filamentation and stress resistance in *C. albicans* or *C. glabrata* under standard laboratory conditions.

In order to study whether *Ca*Hsp12p is essential for virulence of *C. albicans*, a virulence test of the Ca*hsp12* null mutant was carried out in a Toll deficient *Drosophila* line as previously described by us [7]. There was no significant difference in the survival of flies infected with either the Ca*hsp12* null mutant or its control strain (Figure S6), indicating that deletion of Ca*HSP12* did not affect the virulence of *C. albicans*.

### Overexpression of Ca*HSP12* enhances cell aggregation

**Figure 4:**
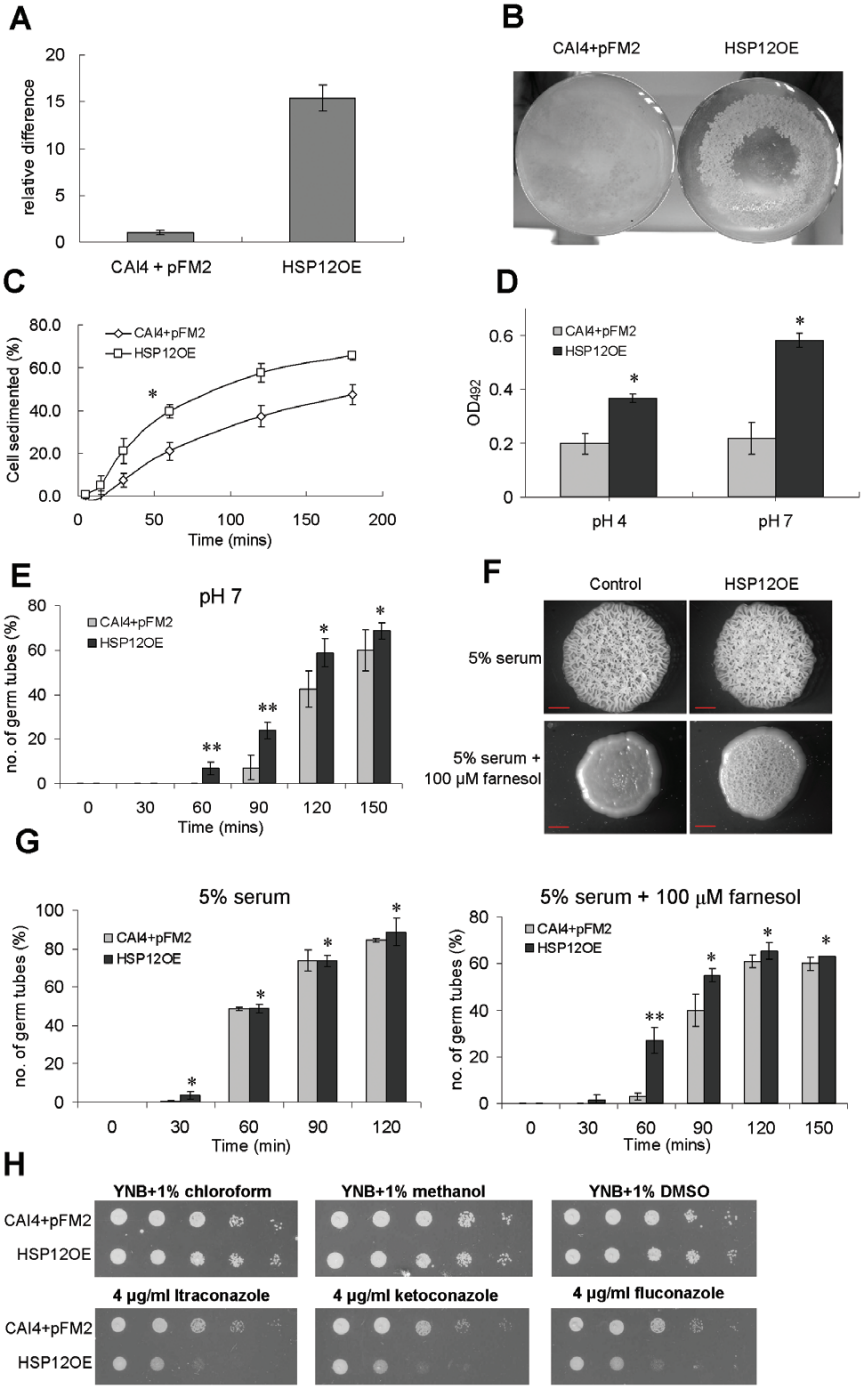
Overexpression of Ca*HSP12* in *C. albicans*. (A) qRT-PCR analysis of the *CaHSP12* transcripts in HSP12OE. The level of transcripts was normalized to *ACT1*. The error bars represent the S.D. of triplicate independent reactions (B) Overexpression of Ca*HSP12* induced cell clumping. The control CAI4+pFM2 and HSP12OE were grown at pH 7. (C) Overexpression of *CaHSP12* promoted cell aggregation which was independent from filamentation. CAI4+pFM2 and HSP12OE were grown at pH 4 for 4 h. Aggregation was then measured. The graphs were plotted by the percentage of cells sedimented against time. Results represent the means of three biological replicates with S.D. *P value<0.05, versus control strain, two-sided unpaired student t-test. (D) Overexpression of Ca*HSP12* enhanced cell adhesion at pH 4 or pH 7. HSP12OE and CAI4+pFM2 were grown on the flat-bottomed 96-well polystyrene plates and incubated at 37°C for 24 h. The adherent cells were quantified using the XTT reduction assay. The error bars were calculated from the S.D. of the triplicates. *P value<0.01, versus control strain, two-sided unpaired student t-test. (E) Overexpression of Ca*HSP12* promoted filamentation at pH 7. The percentage of the germ tube formation was counted every 30 min. The results presented are the means of three biological replicates with the S.D. **P value<0.01, * *P* value>0.05 versus control strains, two-sided unpaired student t-test. (F) Overexpression of Ca*HSP12* impacts on farnesol susceptibility. Cells were spotted onto 5% serum YEPD plates supplemented with or without 100 µm farnesol. Scale bar, 200 µm. (G) CAI4+pFM2 and HSP12OE were incubated in YNB supplemented with 5% serum with or without 100 µM farnesol. Germ tube formation was quantified every 30 min. The error bars were calculated from the S.D. of the triplicates. **P value<0.01, *P value>0.05 versus control strains, two-sided unpaired student t-test. (G) Overexpression of Ca*HSP12* increases susceptibility to azole antifungal drugs. 10-fold dilutions were spotted onto YNB plates containing 4 µg ml^−1^ itraconazole, ketoconazole and fluconzaole. YNB plates supplemented with 1% chloroform, methanol and DMSO act as control.

To explore the function of *Ca*Hsp12 protein further, the gene was expressed under the control of the native *TEF2* promoter generating *C. albicans* HSP12OE. Overexpression was confirmed by using qRT-PCR, showing that the expression of Ca*HSP12* is increased by 15-fold (Figure 4A). The elevated level of Hsp12p in HSP12OE was also seen in Western blot analysis (Figure S7). Although we observed no alterations in stress resistance, including heat shock, osmotic and oxidative stress (Figure S8), HSP12OE was found to form clumps of cells when grown at pH 7 in liquid medium (Figure 4B). Susequently, cell-aggregation was quantified [47] and show that HSP12OE rapidly sedimented to the bottom of the cuvettes if grown at pH 7 and/or 5.5% CO_2_ (Figure S9). Since high pH and CO_2_ are conditions which promote *C. albicans* filamentation [3], [4], [6], [7], [40] we investigated if the cells continue to aggregate at pH 4, a condition where *C. albicans* did not filament. Under this condition HSP12OE settled quicker than the control (Figure 4C), demonstrating that cell aggregation was not secondary to filamentation. Cell adhesion of HSP12OE was also tested using the microtitre plate cell adhesion XTT reduction assay [48], [49]. This showed that HSP12OE adhesion to plastic is much stronger when compared to the control (Figure 4D). Interestingly, cell adhesion of HSP12OE, but not of the control, was further enhanced at pH 7, suggesting that the overexpressing phenotypes were influenced by the environmental pH.

### Overexpression of *HSP12* desensitizes *C. albicans* to the quorum sensing molecule farnesol and enhances susceptibility to azole antifungal agents

When induced at pH 7 HSP12OE filamented earlier, compared to the control strain (Figure 4E). In fact germ tube formation in HSP12OE started after 60 min as opposed to 90 min in the control strain. Since the quorum-sensing molecule farnesol specifically interferes with the yeast-to-hyphal transition of *C. albicans* [50] we investigated its effect on HSP12OE. In the presence of 100 µM farnesol the formation of rough colonies in the control was completely inhibited but filamentation was only marginally affected in HSP12OE colonies (Figure 4F). When quantified in liquid filamentation assays, 100 µM farnesol reduced early-stage germ tube formation to a lesser degree in HSP12OE when compared to the control strain (Figure 4G). Since serum-induced germ tube formation in HSP12OE was not enhanced (Figure 4G), we directly attribute the observed phenotype to a reduced response to farnesol. Overexpression of *Ca*Hsp12p specifically enhanced susceptibility to the azole antifungal agents itraconazole, ketoconazole and fluconazole (Figure 4H) but not amphotericin B and rapamycin (Figure S8).

## Discussion

Although small heat shock proteins are found in most organisms [51] their functions are still poorly understood. Previously, we have shown that expression of the *C. albicans* small heat shock gene, *HSP12*, is regulated by physiologically levels of CO_2_ and pH [21]. Interestingly these studies revealed that the *S. cerevisiae* orthologue of *HSP12* is unaffected by the change of pH, suggesting different mechanisms of adaptation between the two yeasts [21].

Here we characterize Ca*HSP12* from the fungal pathogen *C. albicans* and show that it has both similar and different features when compared with other fungal species. Ca*HSP12* differs from other fungal species for carrying two different loci of the gene, both of which are transcriptionally expressed. We found that *Ca*Hsp12p and *Sc*Hsp12p are diversely regulated in response to oxidative stress. *C. albicans* has a greater level of resistance to oxidative stress than *S. cerevisiae* [40] which may be due to the fact that it has to cope with oxidative stress when phagocytosed by macrophages. Similar to other heat shock proteins, *Ca*Hsp12p is induced in stationary phase [17]. Previously it has been suggested that an increased degree of environmental stress resistance is correlated to the entry into stationary phase favoring long term viability [52], [53]. *Ca*Hsp12p was strongly induced subsequent to heat shock and a bioinformatics approach revealed several heat shock elements (HSE) in the upstream non-coding region. Nicholls *et al* have shown that the heat shock factor-1 Hsflp is activated under heat shock and required for the expression of heat shock genes, by specifically binding to HSE [54]. In fact microarray analysis showed that Ca*HSP12* is highly up-regulated, in a Hsf1p-dependent manner, in response to heat shock [54]. Additionally, chromatin immunoprecipitation (ChIP) revealed that *Sc*Hsp12p is targeted by Hsf1p in *S. cerevisiae* [55].

We identified E-boxes in the Ca*HSP12* promoter and demonstrated that the transcriptional regulator Efg1p is required not only for *C. albicans* Hsp12p baseline expression in unstressed condition but also under heat and oxidative stress. Previous studies have shown that Efg1p is required for heat stress adaptation, so it is possible that regulation of Hsp12p in response to heat shock is dependent on Efg1p. However, induction of Hsp12p is still found in the *efg1* mutant and additional pathways may be involved in the regulation. Interestingly, Efg1p is not required for the expression of Hsp12p under osmotic stress. This suggests that there are different mechanisms for *C. albicans* in response to different stresses. Hog1p for example is known to play a role in response to osmotic and oxidative stress in *C. albicans* [8]. Unexpectedly, we found *Ca*Hsp12p to be repressed by Hog1p. One could speculate that dephosphorylated Hog1p represses the *Ca*Hsp12p expression whereas stress-mediated phosphorylation of Hog1p can abolish the repression [8].

We found that *Ca*Hsp12p protein is also regulated by changes of environmental CO_2_ and pH confirming our previous results investigating Ca*HSP12* mRNA levels [21]. Furthermore we found *Ca*Hsp12p protein levels to be significantly affected by the quorum-sensing molecule farnesol, which blocks the yeast-to-hyphal transition via the cAMP-dependent signaling cascade [46], [56], [57], [58], [59], confirming results reported by Davis-Hanna *et al.* [24]. Hall *et al.* recently showed that farnesol directly inhibits the adenylyl cyclase, Cyr1p [46], [59] suggesting a link between CaHsp12p expression and farnesol inhibition of Cyr1p. This is consistent with the elevated levels of CaHsp12p found in the *cyr1* mutant. Overexpression of Hsp12p desensitized cells to the effect of farnesol. Although the mechanism of farnesol tolerance is still unclear, Hsp12 protein may have a role in protecting the targets of farnesol or the components of farnesol response pathways [46], [59].

Since ambient pH, CO_2_ and farnesol are all signals which impact on *C. albicans* filamentation, it was hypothesized that the expression of *Ca*Hsp12p is required during morphogenesis. However, deletion of *HSP12* showed that the gene is not essential for *C. albicans* stress resistance, filamentation and virulence. However, *HSP12* overexpression did enhance the early stage of hyphal formation and reduced the effect of farnesol on the inhibition of filamentation. This suggests that *Ca*Hsp12p may have a facilitating role in hyphal formation.

Overexpression of Ca*HSP12* increased the sensitivity of the cells to several azole antifungal drugs. The action of azoles on fungi is mediated by depletion of ergosterol, which results in the alteration of membrane fluidity [60]. In *S. cerevisiae*, *Sc*Hsp12p is known to influence plasma membrane fluidity enhancing the stability of the cell membrane [61]. Overexpression also enhanced cell adhesion. Interestingly the actions of adhesion are mediated by cell wall proteins [1] and *Sc*Hsp12p has been shown to be localized in cell wall [62]. *Ca*Hsp12p has 43% homology to the amino-terminal region of *Sc*Hsp12p, thus it is feasible to speculate that *Ca*Hsp12p is present in the cell wall and as a heat shock protein it may have a role in protection of cell wall proteins. Localization studies of *Ca*Hsp12p *in vivo* in response to stress and during the yeast-to-hyphal transition are required to address this further.

We show the *Ca*Hsp12p is significantly regulated under a wide range of stimuli, but is not essential for *C. albicans* to survive in those conditions. This raises the possibility that other proteins with similar functions may compensate for the inactivation of *Ca*Hsp12p in the Ca*hsp12* null mutant. Our overexpression studies point to the potential role of *Ca*Hsp12p in protecting the targets of farnesol, the cell membrane and cell wall protection. Therefore, identifying protein partners of *Ca*Hsp12p should be of interest and reveal additional information on its biological function.

## Supporting Information

Figure S1 **Expression of** ***Ca*** **Hsp12p in** ***C. albicans*** **mutant strains.** Western blot analysis showing that levels of CaHsp12p remained high in *hog1*, *cyr1*, *tpk1* mutants and unchanged in *tpk2* mutant when heat shocked from 37°C to 45°C, 0.3 M NaCl or 1 mM H_2_O_2_. H: anti-Hsp12p antibody. A: anti-actin antibody (equal protein loading control). RDU: relative densitometry units.
(TIF)

Figure S2 **Deletion of** ***HSP12*** **does not affect growth rate and cell adhesion.** (A) No significant change in the growth rates of the Ca*hsp12* (HSP12KO5) and Cg*hsp12* (Cg12KO) null mutants was observed. The overnight cultures were diluted into the OD_600_ of 0.1 and incubated at 37°C. The OD_600_ of the cells was measured at the indicated time points. The growth curves of strains were plotted in the OD_600_ against time. Triplicate biological experiments have been performed. The error bars represent the S.D. of the triplicate independent experiments. (B) The Ca*hsp12* and the Cg*hsp12* null mutant displayed the same ability of cell adhesion as controls in the XTT reduction assay. The strains were grown on the flat-bottomed 96-well polystyrene plates and incubated at 37°C for 24 h. The adherent cells were quantified using the XTT reduction assay. The results presented are the means of three biological replicates with standard derivation. *P value>0.05 versus controls, two-sided unpaired student t-test.
(TIF)

Figure S3 **Ca** ***HSP12*** **is not essential for** ***C. albicans*** **in resistance to stresses and antifungal drugs.** Overnight cultures were diluted in YEPD liquid to an OD_600_ of 2. For heat shock test, the cells were heated at 55°C for 2 min and 10-fold dilutions of the cells were spotted onto YEPD. For other stress tests, the cells at 10-fold dilutions were spotted onto YNB plates containing stress or antifungal agents as indicated. The cultural plates were incubated at 37°C for 24 h. The YNB plates supplemented with 1% chloroform, methanol and DMSO act as control of itraconazole, ketoconazole and fluconazole which were dissolved in chloroform, methanol and DMSO.
(TIF)

Figure S4 **Deletion of** ***CgHSP12*** **did not affect resistance to stress and antifungal drugs.** The overnight cultures were diluted in YEPD liquid to an OD_600_ of 2, and heat shock at 55°C for 2 min. The cells at 10-fold dilutions were spotted onto YEPD plates and incubated at 37°C for 24 h. For other stress tests, the cells at 10-fold dilutions were spotted onto YNB plates containing stress or antifungal agents as indicated. The cultural plates were incubated at 37°C for 24 h. The YNB plates supplemented with 1% chloroform, methanol and DMSO act as control of itraconazole, ketoconazole and fluconazole.
(TIF)

Figure S5 **Deletion of Ca** ***HSP12*** **does not interfere with filamentation at pH 7 in 5.5% CO_2_.** The Ca*hsp12* deletion strain and its controls were incubated in YNB minimal medium at pH 7 in 5.5% CO_2_ at 37°C. The cell morphology of the strains was observed by a light microscopy. The percentage of the germ tube formation was counted under the microscopy every 30 min. The germ tube formation of the Ca*hsp12* null mutant had no significant difference to the controls. Results presented are the means of three biological replicates with standard derivation. *P* value>0.1 versus controls, two-sided unpaired student t-test.
(TIF)

Figure S6 **Deletion of Ca** ***HSP12*** **does not influence the virulence of** ***C. albicans*** **in the Toll mutant fruit fly.** 15 flies per experimental group were injected with the *C. albicans* strains. The flies were then incubated at 30°C for 40 h. The numbers of the living flies were counted at the indicated time. The results are calculated from the means of three biological replicates with the standard derivations. *P value>0.1, versus control strains (BWT or HSP12C), two-sided unpaired student t-test.
(TIF)

Figure S7 **Western blot analysis of the CaHsp12p expression in HSP12OE.** CaHsp12p was expressed higher in the HSP12OE when compared to wild-type. The blot was hybridised with the anti-Hsp12p antibody and the anti-actin antibody, served as the control for equal protein loading as described in text S1.
(TIF)

Figure S8 **Overexpression of** ***CaHSP12*** **does not affect growth under stresses and exposure to antifungal agents.** For the heat shock assay, the overnight cultures were diluted to OD_600_ of 2 and shifted to 55°C for 2 min. The 10-fold serial dilutions of the heat shock cells were spotted onto YEPD plates and incubated at 37°C for 24 h. For other stress studies, the overnight cultures at the OD_600_ of 2.0 were diluted 10-fold serially. The dilutions (5 µl) were spotted onto YNB plates supplemented with stress and antifungal agents as indicated. The plates were incubated at 37°C for 24 h.
(TIF)

Figure S9 **Overexpression of Ca** ***HSP12*** **promotes cell aggregation at pH 7 in air or 5.5% CO_2_.** The strains were grown at (A) pH 7 in air; (B) pH 7 in 5.5% CO_2_. Total 1 ml of the culture was settled to the bottom of the cuvettes. The OD_600_ corresponding to the cells at the upper part of the cuvettes was measured at the time points indicated. The graphs were plotted by the percentage of cell sedimented against time. Results represent the means of three biological replicates with standard derivation. *P value<0.05, versus control strain, two-sided unpaired student t-test.
(TIF)

Text S1 **Supplemental** **Materials and Methods** **.**
(DOC)

## References

[1] Mavor AL, Thewes S, Hube B (2005) Systemic fungal infections caused by *Candida* species: epidemiology, infection process and virulence attributes. Curr Drug Targets 6: 863–874. 16375670 10.2174/138945005774912735

[2] Hosking S (1999) Fungi as animal pathogens. In: Oliver RP, Schweizer M, editors. Molecular fungal biology. Cambridge: Cambridge University Press. pp. 322–340.

[3] Mühlschlegel FA, Fonzi WA (1997) *PHR2* of *Candida albicans* encodes a functional homolog of the pH-regulated gene *PHR1* with an inverted pattern of pH-dependent expression. Mol Cell Biol 17: 5960–5967. 9315654 10.1128/mcb.17.10.5960 PMC232444

[4] El Barkani A, Kurzai O, Fonzi WA, Ramon A, Porta A, et al (2000) Dominant active alleles of *RIM101* (*PRR2*) bypass the pH restriction on filamentation of *Candida albicans* . Mol Cell Biol 20: 4635–4647. 10848590 10.1128/mcb.20.13.4635-4647.2000 PMC85869

[5] Cottier F, Raymond M, Kurzai O, Bolstad M, Leewattanapasuk W, et al (2012) The bZIP transcription factor Rca1p is a central regulator of a novel CO_2_ sensing pathway in yeast. PLoS Pathog 8: e1002485. 22253597 10.1371/journal.ppat.1002485 PMC3257301

[6] Klengel T, Liang WJ, Chaloupka J, Ruoff C, Schroppel K, et al (2005) Fungal adenylyl cyclase integrates CO_2_ sensing with cAMP signaling and virulence. Curr Biol 15: 2021–2026. 16303561 10.1016/j.cub.2005.10.040 PMC3646525

[7] Hall RA, De Sordi L, Maccallum DM, Topal H, Eaton R, et al (2010) CO_2_ acts as a signalling molecule in populations of the fungal pathogen *Candida albicans* . PLoS Pathog 6: e1001193. 21124988 10.1371/journal.ppat.1001193 PMC2987819

[8] Smith DA, Nicholls S, Morgan BA, Brown AJ, Quinn J (2004) A conserved stress-activated protein kinase regulates a core stress response in the human pathogen *Candida albicans* . Mol Biol Cell 15: 4179–4190. 15229284 10.1091/mbc.E04-03-0181 PMC515350

[9] Whiteway M, Bachewich C (2007) Morphogenesis in *Candida albicans* . Annu Rev Microbiol 61: 529–553. 17506678 10.1146/annurev.micro.61.080706.093341 PMC4452225

[10] Csank C, Schroppel K, Leberer E, Harcus D, Mohamed O, et al (1998) Roles of the *Candida albicans* mitogen-activated protein kinase homolog, Cek1p, in hyphal development and systemic candidiasis. Infect Immun 66: 2713–2721. 9596738 10.1128/iai.66.6.2713-2721.1998 PMC108260

[11] Davis D, Wilson RB, Mitchell AP (2000) *RIM101*-dependent and-independent pathways govern pH responses in *Candida albicans* . Mol Cell Biol 20: 971–978. 10629054 10.1128/mcb.20.3.971-978.2000 PMC85214

[12] Kadosh D, Johnson AD (2005) Induction of the *Candida albicans* filamentous growth program by relief of transcriptional repression: a genome-wide analysis. Mol Biol Cell 16: 2903–2912. 15814840 10.1091/mbc.E05-01-0073 PMC1142434

[13] Burnie JP, Carter TL, Hodgetts SJ, Matthews RC (2006) Fungal heat-shock proteins in human disease. FEMS Microbiol Rev 30: 53–88. 16438680 10.1111/j.1574-6976.2005.00001.x

[14] Praekelt UM, Meacock PA (1990) *HSP12*, a new small heat shock gene of *Saccharomyces cerevisiae*: analysis of structure, regulation and function. Mol Gen Genet 223: 97–106. 2175390 10.1007/BF00315801

[15] de Jong WW, Leunissen JA, Voorter CE (1993) Evolution of the alpha-crystallin/small heat-shock protein family. Mol Biol Evol 10: 103–126. 8450753 10.1093/oxfordjournals.molbev.a039992

[16] Garstel M, Vajdy E, Buchner J (1997) The small Hsps - an overview. In: Gething M, editor. Guidebook to the molecular chaperones and protein-folding catalysts. Oxford: Oxford University Press. pp. 269–272.

[17] Panaretou B, Piper PW (1992) The plasma membrane of yeast acquires a novel heat-shock protein (hsp30) and displays a decline in proton-pumping ATPase levels in response to both heat shock and the entry to stationary phase. Eur J Biochem 206: 635–640. 1535043 10.1111/j.1432-1033.1992.tb16968.x

[18] Carmelo V, Sa-Correia I (1997) *HySP26* gene transcription is strongly induced during *Saccharomyces cerevisiae* growth at low pH. FEMS Microbiol Lett 149: 85–88. 9103979 10.1111/j.1574-6968.1997.tb10312.x

[19] Bentley NJ, Fitch IT, Tuite MF (1992) The small heat-shock protein Hsp26 of *Saccharomyces cerevisiae* assembles into a high molecular weight aggregate. Yeast 8: 95–106. 1561840 10.1002/yea.320080204

[20] Liu TT, Lee RE, Barker KS, Wei L, Homayouni R, et al (2005) Genome-wide expression profiling of the response to azole, polyene, echinocandin, and pyrimidine antifungal agents in *Candida albicans* . Antimicrob Agents Chemother 49: 2226–2236. 15917516 10.1128/AAC.49.6.2226-2236.2005 PMC1140538

[21] Sheth CC, Mogensen EG, Fu MS, Blomfield IC, Mühlschlegel FA (2008) *Candida albicans HSP12* is co-regulated by physiological CO_2_ and pH. Fungal Genet Biol 45: 1075–1080. 18487064 10.1016/j.fgb.2008.04.004

[22] Enjalbert B, Nantel A, Whiteway M (2003) Stress-induced gene expression in *Candida albicans*: absence of a general stress response. Mol Biol Cell 14: 1460–1467. 12686601 10.1091/mbc.E02-08-0546 PMC153114

[23] Enjalbert B, Smith DA, Cornell MJ, Alam I, Nicholls S, et al (2006) Role of the Hog1 stress-activated protein kinase in the global transcriptional response to stress in the fungal pathogen *Candida albicans* . Mol Biol Cell 17: 1018–1032. 16339080 10.1091/mbc.E05-06-0501 PMC1356608

[24] Davis-Hanna A, Piispanen AE, Stateva LI, Hogan DA (2008) Farnesol and dodecanol effects on the *Candida albicans* Ras1-cAMP signalling pathway and the regulation of morphogenesis. Mol Microbiol 67: 47–62. 18078440 10.1111/j.1365-2958.2007.06013.x PMC3782305

[25] Setiadi ER, Doedt T, Cottier F, Noffz C, Ernst JF (2006) Transcriptional response of *Candida albicans* to hypoxia: linkage of oxygen sensing and Efg1p-regulatory networks. J Mol Biol 361: 399–411. 16854431 10.1016/j.jmb.2006.06.040

[26] Coste AT, Karababa M, Ischer F, Bille J, Sanglard D (2004) *TAC1*, transcriptional activator of *CDR* genes, is a new transcription factor involved in the regulation of *Candida albicans* ABC transporters *CDR1* and *CDR2* . Eukaryot Cell 3: 1639–1652. 15590837 10.1128/EC.3.6.1639-1652.2004 PMC539021

[27] Karababa M, Coste AT, Rognon B, Bille J, Sanglard D (2004) Comparison of gene expression profiles of *Candida albicans* azole-resistant clinical isolates and laboratory strains exposed to drugs inducing multidrug transporters. Antimicrob Agents Chemother 48: 3064–3079. 15273122 10.1128/AAC.48.8.3064-3079.2004 PMC478486

[28] Thewes S, Kretschmar M, Park H, Schaller M, Filler SG, et al (2007) In vivo and ex vivo comparative transcriptional profiling of invasive and non-invasive *Candida albicans* isolates identifies genes associated with tissue invasion. Mol Microbiol 63: 1606–1628. 17367383 10.1111/j.1365-2958.2007.05614.x

[29] Nantel A, Dignard D, Bachewich C, Harcus D, Marcil A, et al (2002) Transcription profiling of *Candida albicans* cells undergoing the yeast-to-hyphal transition. Mol Biol Cell 13: 3452–3465. 12388749 10.1091/mbc.E02-05-0272 PMC129958

[30] Lan CY, Rodarte G, Murillo LA, Jones T, Davis RW, et al (2004) Regulatory networks affected by iron availability in *Candida albicans* . Mol Microbiol 53: 1451–1469. 15387822 10.1111/j.1365-2958.2004.04214.x

[31] Vermitsky JP, Earhart KD, Smith WL, Homayouni R, Edlind TD, et al (2006) Pdr1 regulates multidrug resistance in *Candida glabrata*: gene disruption and genome-wide expression studies. Mol Microbiol 61: 704–722. 16803598 10.1111/j.1365-2958.2006.05235.x

[32] Maeng S, Ko YJ, Kim GB, Jung KW, Floyd A, et al (2010) Comparative transcriptome analysis reveals novel roles of the Ras and cyclic AMP signaling pathways in environmental stress response and antifungal drug sensitivity in Cryptococcus neoformans. Eukaryot Cell 9: 360–378. 20097740 10.1128/EC.00309-09 PMC2837985

[33] Fonzi WA, Irwin MY (1993) Isogenic strain construction and gene mapping in *Candida albicans* . Genetics 134: 717–728. 8349105 10.1093/genetics/134.3.717 PMC1205510

[34] Wilson RB, Davis D, Mitchell AP (1999) Rapid hypothesis testing with *Candida albicans* through gene disruption with short homology regions. J Bacteriol 181: 1868–1874. 10074081 10.1128/jb.181.6.1868-1874.1999 PMC93587

[35] Wendland J (2003) PCR-based methods facilitate targeted gene manipulations and cloning procedures. Curr Genet 44: 115–123. 12928752 10.1007/s00294-003-0436-x

[36] Dennison PM, Ramsdale M, Manson CL, Brown AJ (2005) Gene disruption in *Candida albicans* using a synthetic, codon-optimised Cre-loxP system. Fungal Genet Biol 42: 737–748. 16043373 10.1016/j.fgb.2005.05.006

[37] Weig M, Haynes K, Rogers TR, Kurzai O, Frosch M, et al (2001) A *GAS*-like gene family in the pathogenic fungus *Candida glabrata* . Microbiology 147: 2007–2019. 11495979 10.1099/00221287-147-8-2007

[38] Kitada K, Yamaguchi E, Arisawa M (1995) Cloning of the *Candida glabrata TRP1* and *HIS3* genes, and construction of their disruptant strains by sequential integrative transformation. Gene 165: 203–206. 8522176 10.1016/0378-1119(95)00552-h

[39] Petko L, Lindquist S (1986) Hsp26 is not required for growth at high temperatures, nor for thermotolerance, spore development, or germination. Cell 45: 885–894. 3518952 10.1016/0092-8674(86)90563-5

[40] Jamieson DJ, Rivers SL, Stephen DW (1994) Analysis of *Saccharomyces cerevisiae* proteins induced by peroxide and superoxide stress. Microbiology 140 (Pt 12) 3277–3283. 7881546 10.1099/13500872-140-12-3277

[41] Varela JC, Praekelt UM, Meacock PA, Planta RJ, Mager WH (1995) The *Saccharomyces cerevisiae HSP12* gene is activated by the high-osmolarity glycerol pathway and negatively regulated by protein kinase A. Mol Cell Biol 15: 6232–6245. 7565776 10.1128/mcb.15.11.6232 PMC230875

[42] Cowen LE, Nantel A, Whiteway MS, Thomas DY, Tessier DC, et al (2002) Population genomics of drug resistance in *Candida albicans* . Proc Natl Acad Sci U S A 99: 9284–9289. 12089321 10.1073/pnas.102291099 PMC123132

[43] Bockmühl DP, Krishnamurthy S, Gerads M, Sonneborn A, Ernst JF (2001) Distinct and redundant roles of the two protein kinase A isoforms Tpk1p and Tpk2p in morphogenesis and growth of *Candida albicans* . Mol Microbiol 42: 1243–1257. 11886556 10.1046/j.1365-2958.2001.02688.x

[44] Eckert SE, Heinz WJ, Zakikhany K, Thewes S, Haynes K, et al (2007) *PGA4*, a *GAS* homologue from *Candida albicans*, is up-regulated early in infection processes. Fungal Genet Biol 44: 368–377. 17257864 10.1016/j.fgb.2006.12.006

[45] Braun BR, Johnson AD (1997) Control of filament formation in *Candida albicans* by the transcriptional repressor *TUP1* . Science 277: 105–109. 9204892 10.1126/science.277.5322.105

[46] Hall RA, Turner KJ, Chaloupka J, Cottier F, De Sordi L, et al (2011) The quorum-sensing molecules farnesol/homoserine lactone and dodecanol operate via distinct modes of action in *Candida albicans* . Eukaryot Cell 10: 1034–1042. 21666074 10.1128/EC.05060-11 PMC3165441

[47] Eboigbodin KE, Biggs CA (2008) Characterization of the extracellular polymeric substances produced by Escherichia coli using infrared spectroscopic, proteomic, and aggregation studies. Biomacromolecules 9: 686–695. 18186609 10.1021/bm701043c

[48] Jin Y, Yip HK, Samaranayake YH, Yau JY, Samaranayake LP (2003) Biofilm-forming ability of *Candida albicans* is unlikely to contribute to high levels of oral yeast carriage in cases of human immunodeficiency virus infection. J Clin Microbiol 41: 2961–2967. 12843027 10.1128/JCM.41.7.2961-2967.2003 PMC165379

[49] Hiller E, Heine S, Brunner H, Rupp S (2007) *Candida albicans* Sun41p, a putative glycosidase, is involved in morphogenesis, cell wall biogenesis, and biofilm formation. Eukaryot Cell 6: 2056–2065. 17905924 10.1128/EC.00285-07 PMC2168408

[50] Hornby JM, Jensen EC, Lisec AD, Tasto JJ, Jahnke B, et al (2001) Quorum sensing in the dimorphic fungus *Candida albicans* is mediated by farnesol. Appl Environ Microbiol 67: 2982–2992. 11425711 10.1128/AEM.67.7.2982-2992.2001 PMC92970

[51] Haslbeck M, Franzmann T, Weinfurtner D, Buchner J (2005) Some like it hot: the structure and function of small heat-shock proteins. Nat Struct Mol Biol 12: 842–846. 16205709 10.1038/nsmb993

[52] Sanchez Y, Taulien J, Borkovich KA, Lindquist S (1992) Hsp104 is required for tolerance to many forms of stress. EMBO J 11: 2357–2364. 1600951 10.1002/j.1460-2075.1992.tb05295.x PMC556703

[53] Elliott B, Futcher B (1993) Stress resistance of yeast cells is largely independent of cell cycle phase. Yeast 9: 33–42. 8442385 10.1002/yea.320090105

[54] Nicholls S, Leach MD, Priest CL, Brown AJ (2009) Role of the heat shock transcription factor, Hsf1, in a major fungal pathogen that is obligately associated with warm-blooded animals. Mol Microbiol 74: 844–861. 19818013 10.1111/j.1365-2958.2009.06883.x PMC3675641

[55] Hahn JS, Hu Z, Thiele DJ, Iyer VR (2004) Genome-wide analysis of the biology of stress responses through heat shock transcription factor. Mol Cell Biol 24: 5249–5256. 15169889 10.1128/MCB.24.12.5249-5256.2004 PMC419887

[56] Ramage G, Saville SP, Wickes BL, Lopez-Ribot JL (2002) Inhibition of *Candida albicans* biofilm formation by farnesol, a quorum-sensing molecule. Appl Environ Microbiol 68: 5459–5463. 12406738 10.1128/AEM.68.11.5459-5463.2002 PMC129887

[57] Westwater C, Balish E, Schofield DA (2005) *Candida albicans*-conditioned medium protects yeast cells from oxidative stress: a possible link between quorum sensing and oxidative stress resistance. Eukaryot Cell 4: 1654–1661. 16215173 10.1128/EC.4.10.1654-1661.2005 PMC1265892

[58] Nickerson KW, Atkin AL, Hornby JM (2006) Quorum sensing in dimorphic fungi: farnesol and beyond. Appl Environ Microbiol 72: 3805–3813. 16751484 10.1128/AEM.02765-05 PMC1489610

[59] Hogan DA, Mühlschlegel FA (2011) *Candida albicans* developmental regulation: adenylyl cyclase as a coincidence detector of parallel signals. Curr Opin Microbiol 14: 682–686. 22014725 10.1016/j.mib.2011.09.014

[60] Smriti, Krishnamurthy SS, Prasad R (1999) Membrane fluidity affects functions of Cdr1p, a multidrug ABC transporter of *Candida albicans* . FEMS Microbiol Lett 173: 475–481. 10227177 10.1111/j.1574-6968.1999.tb13541.x

[61] Welker S, Rudolph B, Frenzel E, Hagn F, Liebisch G, et al (2010) Hsp12 is an intrinsically unstructured stress protein that folds upon membrane association and modulates membrane function. Mol Cell 39: 507–520. 20797624 10.1016/j.molcel.2010.08.001

[62] Motshwene P, Karreman R, Kgari G, Brandt W, Lindsey G (2004) LEA (late embryonic abundant)-like protein Hsp 12 (heat-shock protein 12) is present in the cell wall and enhances the barotolerance of the yeast *Saccharomyces cerevisiae* . Biochem J 377: 769–774. 14570591 10.1042/BJ20031301 PMC1223906

[63] Brachmann CB, Davies A, Cost GJ, Caputo E, Li J, et al (1998) Designer deletion strains derived from *Saccharomyces cerevisiae* S288C: a useful set of strains and plasmids for PCR-mediated gene disruption and other applications. Yeast 14: 115–132. 9483801 10.1002/(SICI)1097-0061(19980130)14:2<115::AID-YEA204>3.0.CO;2-2

