# Supplementary figures and images for: Functional Characterization of the Small Heat Shock Protein Hsp12p from Candida albicans
Source: PLoS One. 2012 Aug 7;7(8):e42894. doi: 10.1371/journal.pone.0042894 (PMC3413664; doi:10.1371/journal.pone.0042894)

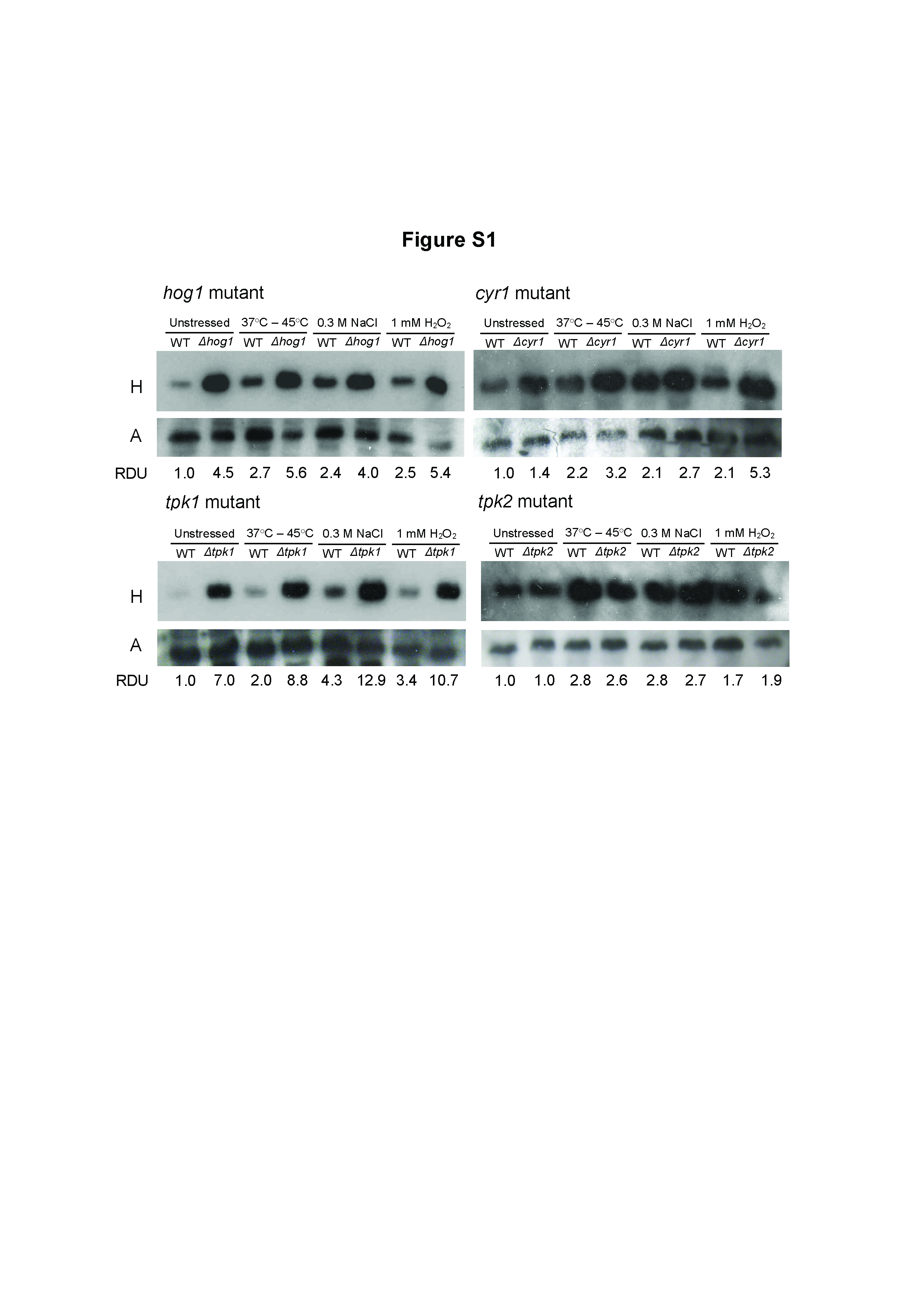

Supplement: Figure S1 — Expression of Ca Hsp12p in C. albicans mutant strains. Western blot analysis showing that levels of CaHsp12p remained high in hog1, cyr1, tpk1 mutants and unchanged in tpk2 mutant when heat shocked from 37°C to 45°C, 0.3 M NaCl or 1 mM H2O2. H: anti-Hsp12p antibody. A: anti-actin antibody (equal protein loading control). RDU: relative densitometry units. (TIF) [file pone.0042894.s001.tif]

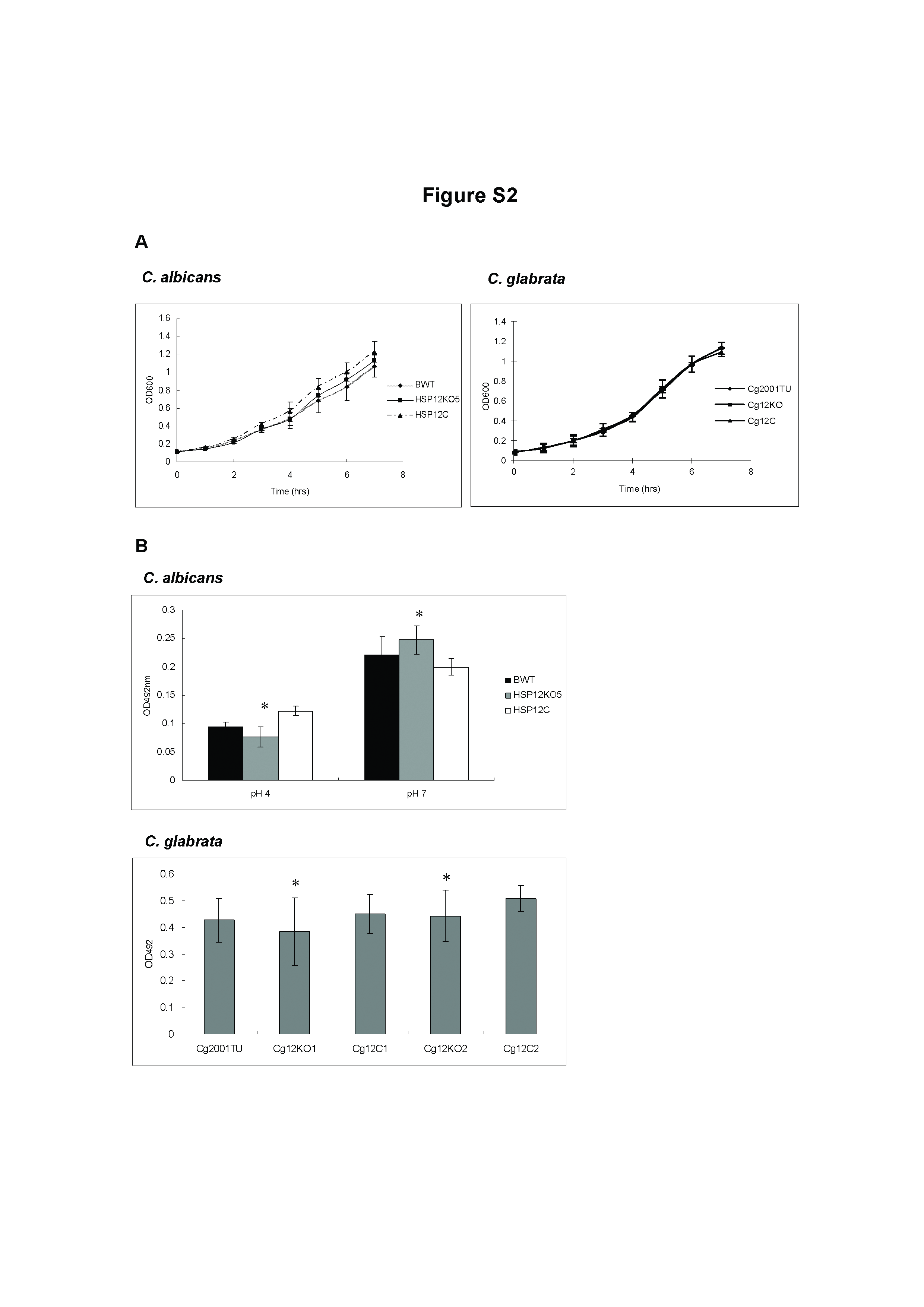

Supplement: Figure S2 — Deletion of HSP12 does not affect growth rate and cell adhesion. (A) No significant change in the growth rates of the Cahsp12 (HSP12KO5) and Cghsp12 (Cg12KO) null mutants was observed. The overnight cultures were diluted into the OD600 of 0.1 and incubated at 37°C. The OD600 of the cells was measured at the indicated time points. The growth curves of strains were plotted in the OD600 against time. Triplicate biological experiments have been performed. The error bars represent the S.D. of the triplicate independent experiments. (B) The Cahsp12 and the Cghsp12 null mutant displayed the same ability of cell adhesion as controls in the XTT reduction assay. The strains were grown on the flat-bottomed 96-well polystyrene plates and incubated at 37°C for 24 h. The adherent cells were quantified using the XTT reduction assay. The results presented are the means of three biological replicates with standard derivation. *P value>0.05 versus controls, two-sided unpaired student t-test. (TIF) [file pone.0042894.s002.tif]

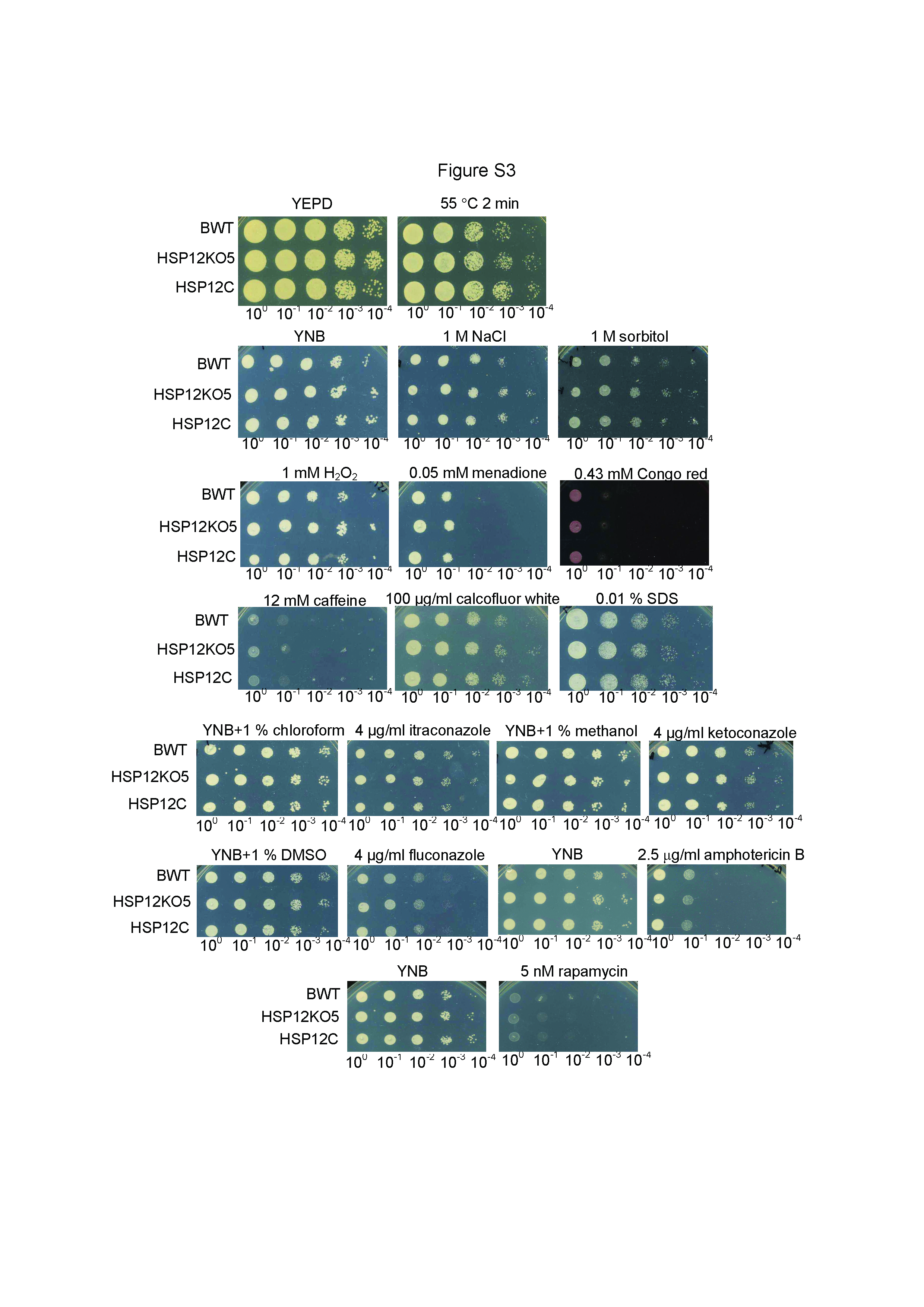

Supplement: Figure S3 — Ca HSP12 is not essential for C. albicans in resistance to stresses and antifungal drugs. Overnight cultures were diluted in YEPD liquid to an OD600 of 2. For heat shock test, the cells were heated at 55°C for 2 min and 10-fold dilutions of the cells were spotted onto YEPD. For other stress tests, the cells at 10-fold dilutions were spotted onto YNB plates containing stress or antifungal agents as indicated. The cultural plates were incubated at 37°C for 24 h. The YNB plates supplemented with 1% chloroform, methanol and DMSO act as control of itraconazole, ketoconazole and fluconazole which were dissolved in chloroform, methanol and DMSO. (TIF) [file pone.0042894.s003.tif]

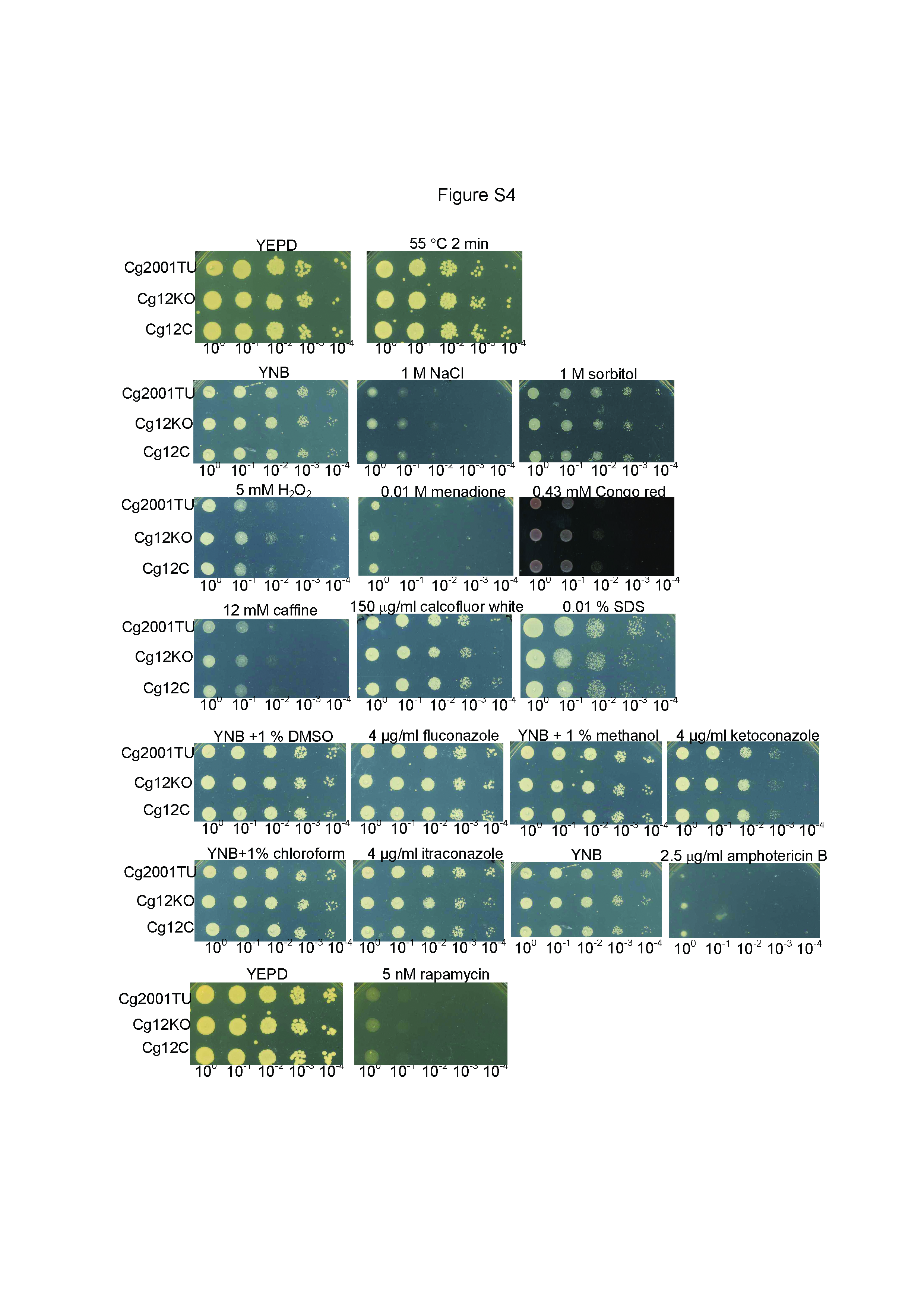

Supplement: Figure S4 — Deletion of CgHSP12 did not affect resistance to stress and antifungal drugs. The overnight cultures were diluted in YEPD liquid to an OD600 of 2, and heat shock at 55°C for 2 min. The cells at 10-fold dilutions were spotted onto YEPD plates and incubated at 37°C for 24 h. For other stress tests, the cells at 10-fold dilutions were spotted onto YNB plates containing stress or antifungal agents as indicated. The cultural plates were incubated at 37°C for 24 h. The YNB plates supplemented with 1% chloroform, methanol and DMSO act as control of itraconazole, ketoconazole and fluconazole. (TIF) [file pone.0042894.s004.tif]

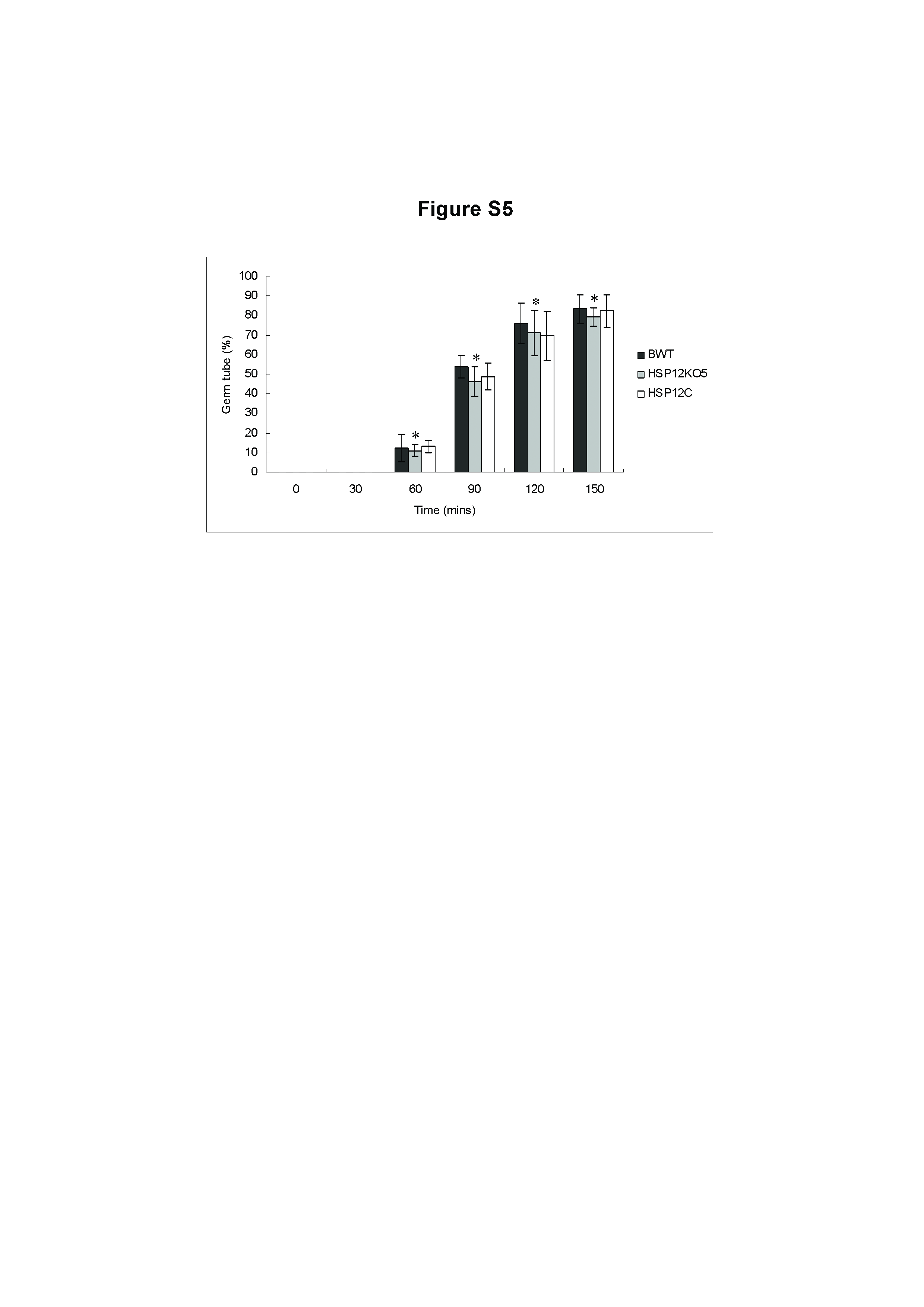

Supplement: Figure S5 — Deletion of Ca HSP12 does not interfere with filamentation at pH 7 in 5.5% CO2. The Cahsp12 deletion strain and its controls were incubated in YNB minimal medium at pH 7 in 5.5% CO2 at 37°C. The cell morphology of the strains was observed by a light microscopy. The percentage of the germ tube formation was counted under the microscopy every 30 min. The germ tube formation of the Cahsp12 null mutant had no significant difference to the controls. Results presented are the means of three biological replicates with standard derivation. P value>0.1 versus controls, two-sided unpaired student t-test. (TIF) [file pone.0042894.s005.tif]

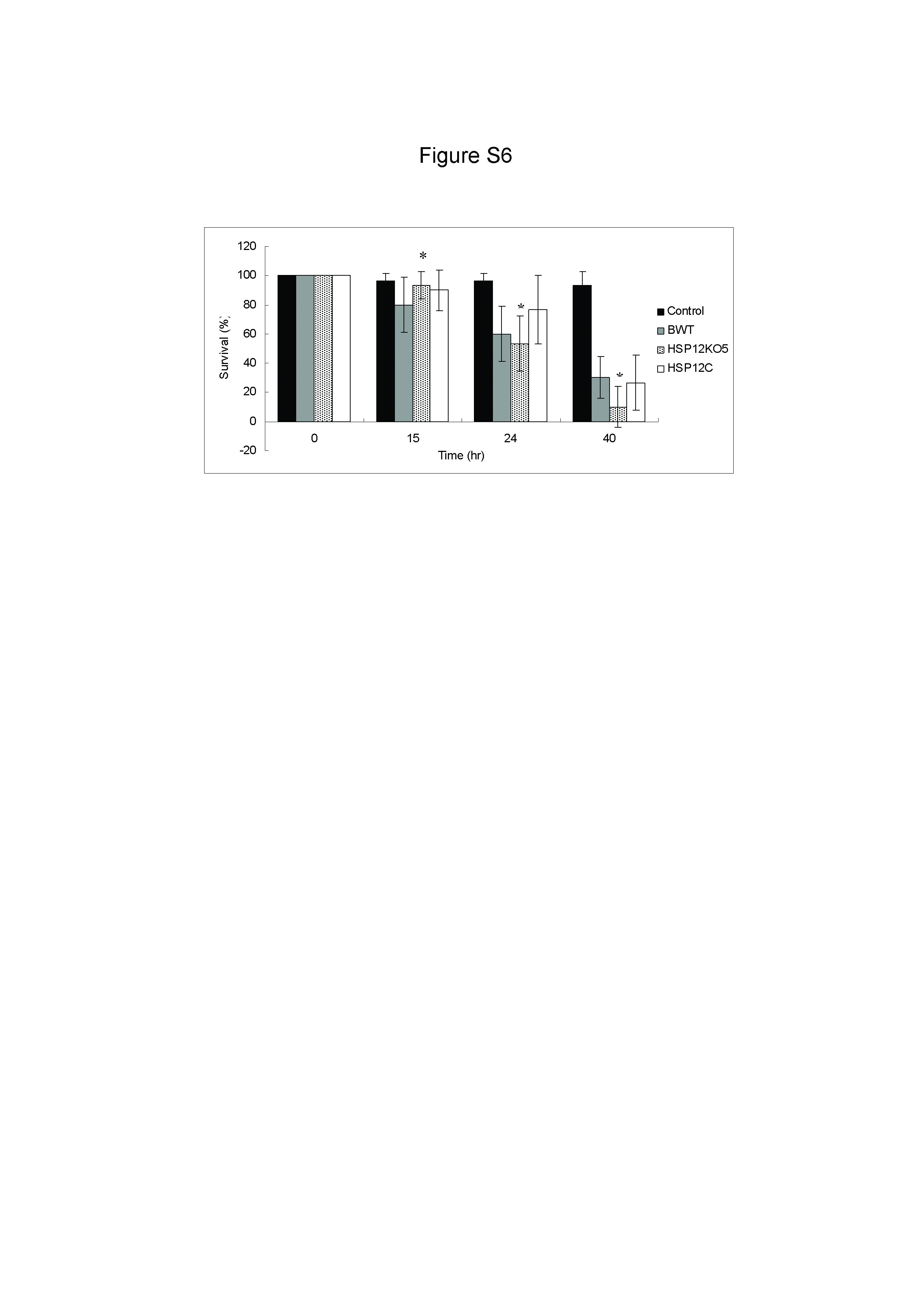

Supplement: Figure S6 — Deletion of Ca HSP12 does not influence the virulence of C. albicans in the Toll mutant fruit fly. 15 flies per experimental group were injected with the C. albicans strains. The flies were then incubated at 30°C for 40 h. The numbers of the living flies were counted at the indicated time. The results are calculated from the means of three biological replicates with the standard derivations. *P value>0.1, versus control strains (BWT or HSP12C), two-sided unpaired student t-test. (TIF) [file pone.0042894.s006.tif]

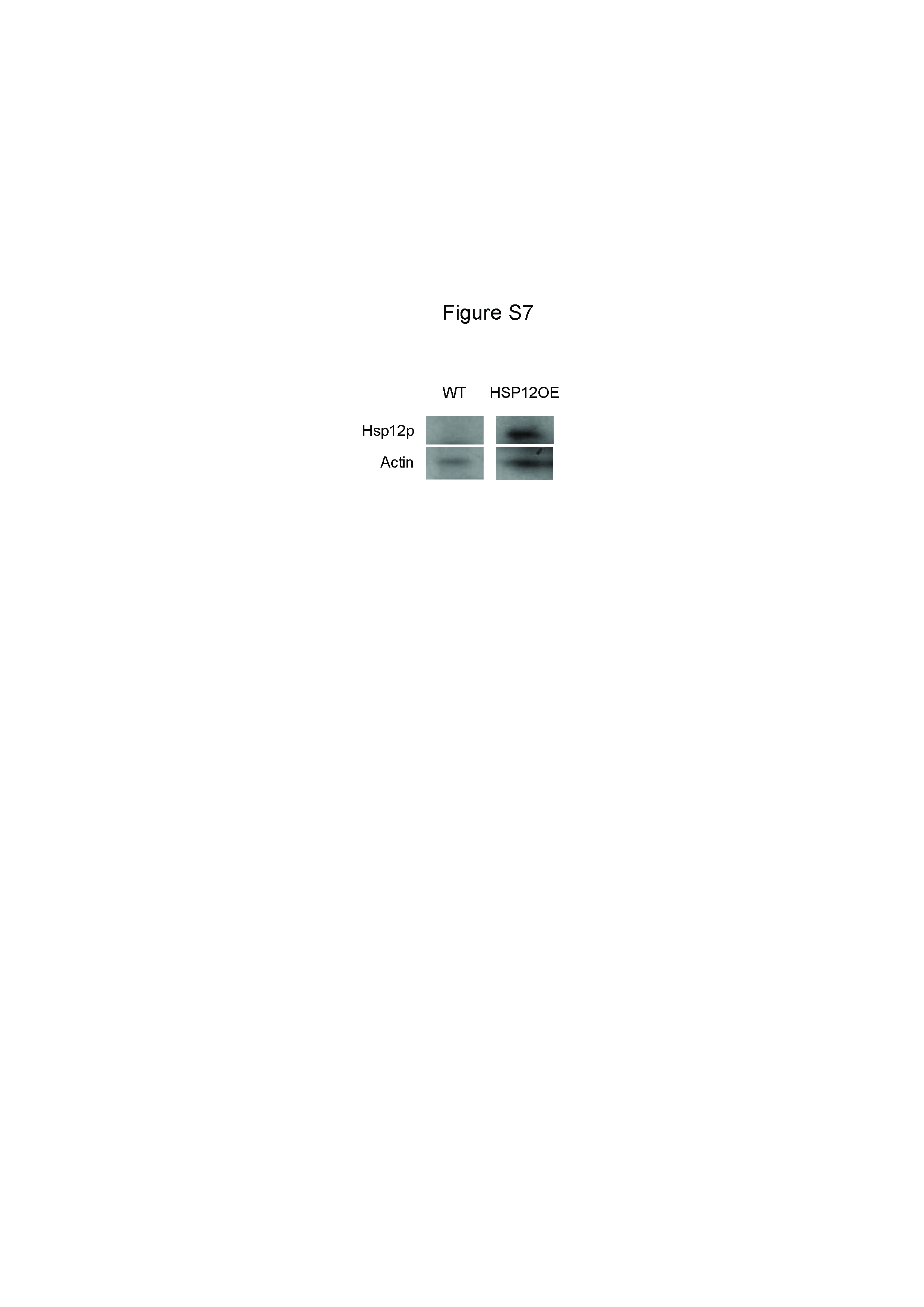

Supplement: Figure S7 — Western blot analysis of the CaHsp12p expression in HSP12OE. CaHsp12p was expressed higher in the HSP12OE when compared to wild-type. The blot was hybridised with the anti-Hsp12p antibody and the anti-actin antibody, served as the control for equal protein loading as described in text S1. (TIF) [file pone.0042894.s007.tif]

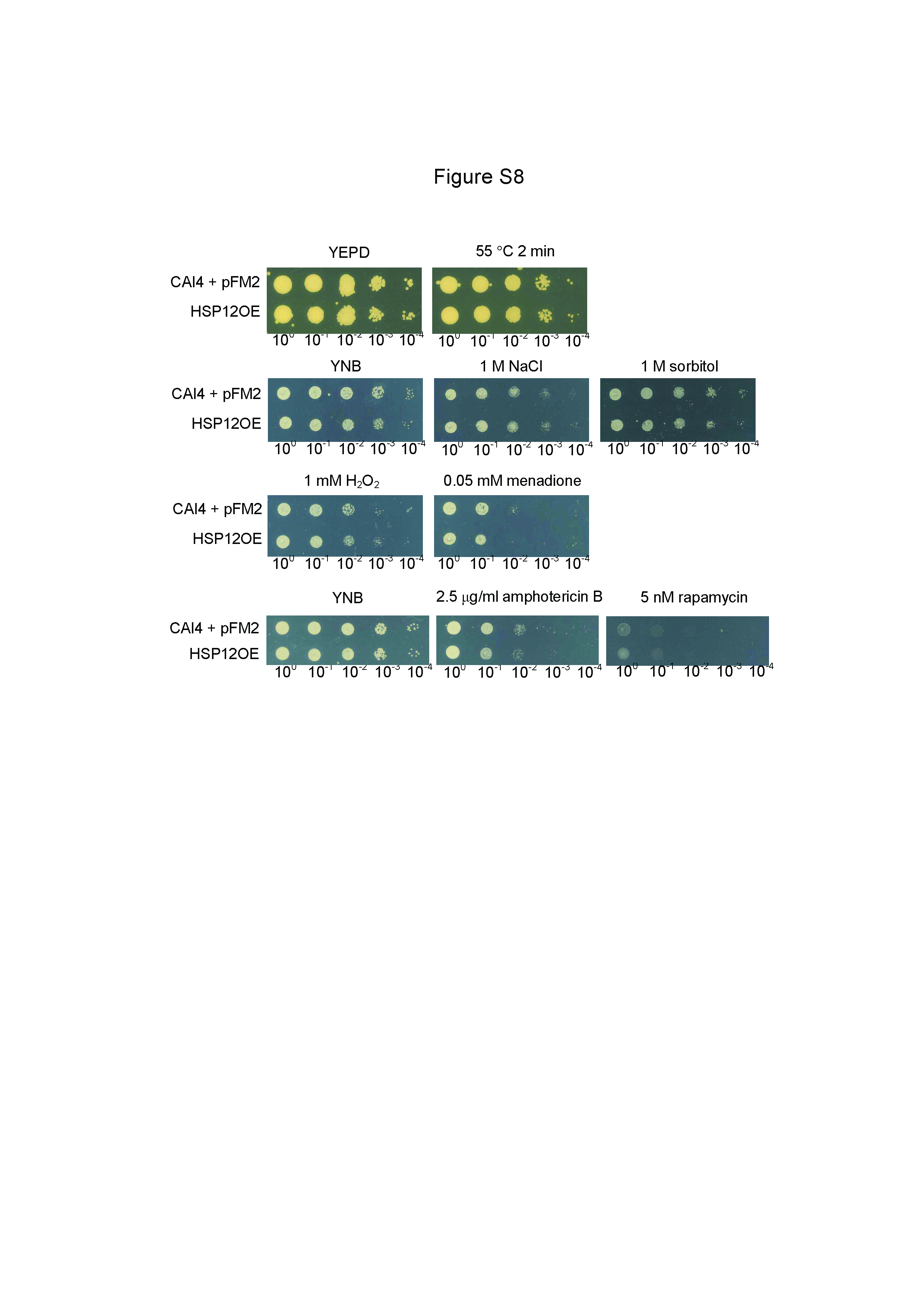

Supplement: Figure S8 — Overexpression of CaHSP12 does not affect growth under stresses and exposure to antifungal agents. For the heat shock assay, the overnight cultures were diluted to OD600 of 2 and shifted to 55°C for 2 min. The 10-fold serial dilutions of the heat shock cells were spotted onto YEPD plates and incubated at 37°C for 24 h. For other stress studies, the overnight cultures at the OD600 of 2.0 were diluted 10-fold serially. The dilutions (5 µl) were spotted onto YNB plates supplemented with stress and antifungal agents as indicated. The plates were incubated at 37°C for 24 h. (TIF) [file pone.0042894.s008.tif]

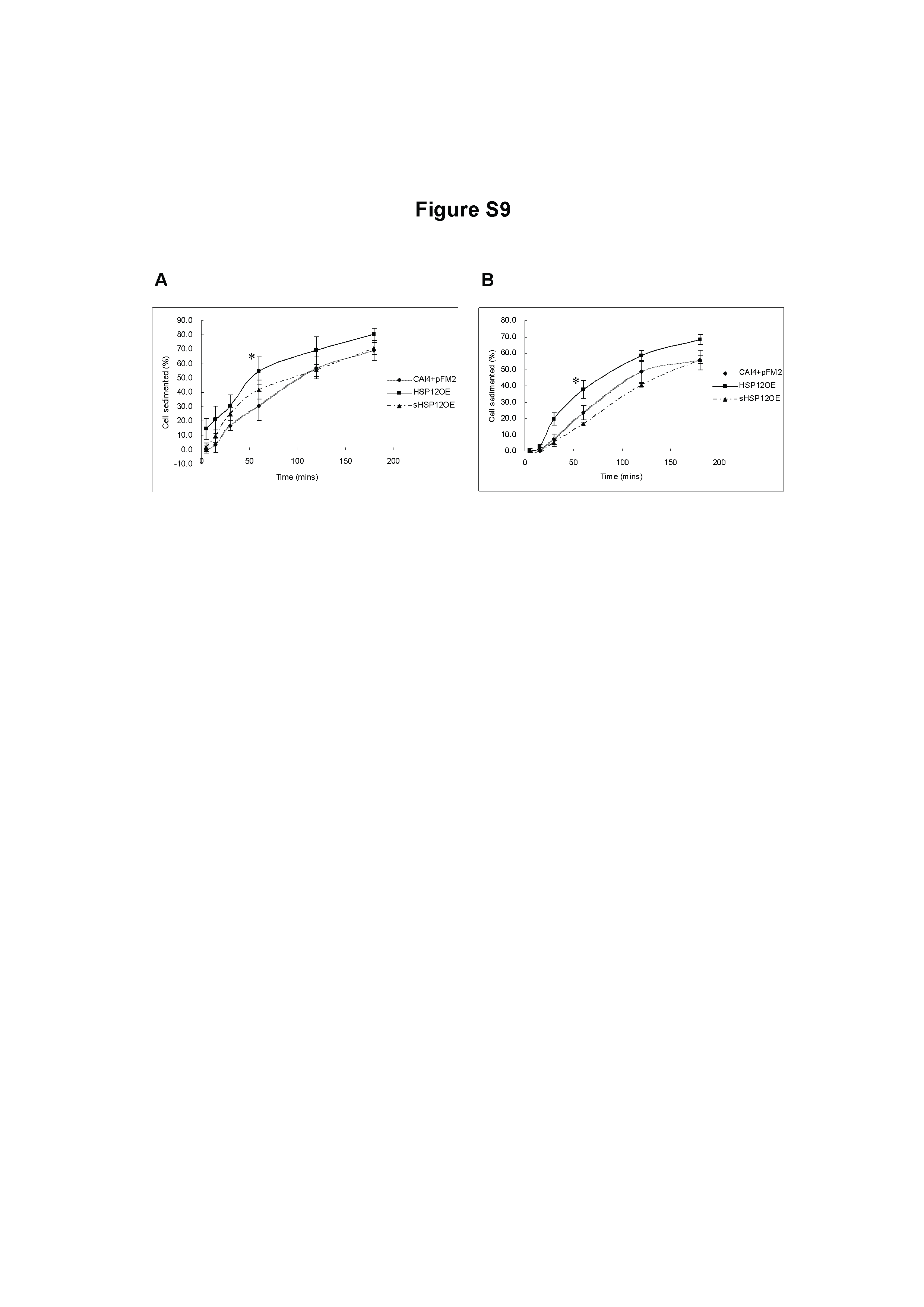

Supplement: Figure S9 — Overexpression of Ca HSP12 promotes cell aggregation at pH 7 in air or 5.5% CO2. The strains were grown at (A) pH 7 in air; (B) pH 7 in 5.5% CO2. Total 1 ml of the culture was settled to the bottom of the cuvettes. The OD600 corresponding to the cells at the upper part of the cuvettes was measured at the time points indicated. The graphs were plotted by the percentage of cell sedimented against time. Results represent the means of three biological replicates with standard derivation. *P value<0.05, versus control strain, two-sided unpaired student t-test. (TIF) [file pone.0042894.s009.tif]
