# Supplementary material for: Functional Characterization of the Small Heat Shock Protein Hsp12p from Candida albicans
Source: PLoS One. 2012 Aug 7;7(8):e42894. doi: 10.1371/journal.pone.0042894 (PMC3413664; doi:10.1371/journal.pone.0042894)
Supplement: Text S1 — Supplemental Materials and Methods . (DOC) [file pone.0042894.s010.doc]

**Supplemental Materials and Methods**

**Construction of *C. albicans* and *C. glabrata* strains**

Disruption cassettes contained the *hisG*-*URA3*-*hisG* or the *HIS1* marker genes flanked by Ca*HSP12*, allowing genomic integration by homologous recombination . First, the Ura-blaster disruption cassette was generated and transformed into BWP17, to obtain the heterozygous mutant strain HSP12KO1. Next, HSP12KO1 was transformed with the *HIS1* disruption cassette to generate the Ca*HSP12a* homozygous mutant strain (HSP12KO2). HSP12KO2 was subsequently treated with 5-fluoroorotic acid (5FOA) to select the *URA3* auxotrophic strain, so the two other alleles of Ca*HSP12b* could be deleted by reusing the Ura-blaster cassette.

To construct the *HIS1* cassette, Ca*HSP12a* (orf19.3160) with 500 bp upstream and 520 bp downstream regions was first amplified by PCR from the *C. albicans* SC5314 genomic DNA using primers HSP12-500F (5’-TCC TCA GCT ACA GTT TCT AC-3’) and HSP12-500R (5’-GCT GTG TTC TAC TAT AG TAC AGA G-3’). The Ca*HSP12* orf was replaced by the *HIS1* marker gene in a *Sac*II-*Bsr*GI fragment. The Ura-blaster cassette was constructed by inserting 500 bp upstream flanking region and 520 bp downstream flanking region of Ca*HSP12* to the *Sac*I-*Kpn*I and *Bam*HI-*Hind*III sites of Ura-blaster plasmid according to standard protocols . The 500 bp upstream flanking region of Ca*HSP12* (orf19.3160 and orf19.4216) was amplified by PCR from the *C. albicans* SC5314 genomic DNA using the primers HSP12_Ura_5F (5’-GGG GGG GAG CTC CGA GGA TAA GCG TTA CCT AGT TTC-3’) and HSP12_Ura_5R (5’-GGG GGG GGT ACC ATC TAC ATA GTG TCG TGT ACA AGG-3’) (*Sac*I and *Kpn*I restriction sites were underlined). The 520 bp downstream flanking region of Ca*HSP12a* (orf19.3160) was amplified by PCR using the primers HSP12_Ura_3F_1 (5’-CCC CCC GGA TCC ATC TAC ATA GTG TCG TGT ACA AG-3’) and HSP12_Ura_3R_1 (5’-CCC CCC AAG CTT GCT GTG TTC TAC TAT AGT ACA GAG-3’). The 520 bp downstream flanking region of Ca*HSP12b* (orf19.4216) was amplified by PCR using the primers HSP12_Ura_3F_2 (5’- CCC CCC GGA TCC ATC TAC ATA GTG TCG TGT ACA AGG -3’) and HSP12_Ura_3R_2 (5’- CCC CCC AAG CTT CGA GGA TAA GCG TTA CCT AGT TTC-3’). A standard lithium acetate transformation protocol was used to introduce the disruption cassettes into the appropriate strains .

Each disruption was confirmed by Southern blot analysis of genomic DNA digested with *Hpa*I. The 1081 bp probe 1, which was located 180 bp downstream of the stop codon of Ca*HSP12a*, was amplified from BWP17 by PCR using primers HSP12-1-SB-F (5’-GGAGACAGTTGACAGACCAGTGATC-3’) and HSP12-1-SB-R (5’-CCATCTAGACTGAGGTCTGCATTTC-3’). Probes 2 (966 bp), located 150 bp downstream of the stop codon of *HSP12b*, was amplified using primers HSP12-2-SB-F (5’- GAGGAAGCATGACAAATGTAGCTAGC-3’) and HSP12-2-SB-R (5’- TTCCAAAGAGCTCGAGCCTCTAC-3’). Deletion of Ca*HSP12* in the null mutant was also confirmed by qRT-PCR.

Reconstitution strains (HSP12C) were constructed by reintegrating a vector CIp30, which contains the *URA3*, *HIS1* and *ARG4* markers, with a wild-type copy of Ca*HSP12* to the *RP10* locus as a standard procedure in *C. albicans* molecular biology . The Ca*HSP12b* locus, which includes the 507 bp Ca*HSP12* ORF, 631 bp putative promoter and 621 bp putative terminator, was PCR amplified using primers HSP12_500G1 (5’- CTTGTCCTCGACATAAACAGCC–3’) and HSP12_500G4 (5’-GCTCAGATGGAAACATGAATACC- 3’). Plasmid Ca*HSP12*-CIp30 was linearized with *Stu*I and transformed into strain HSP12KO4. *Stu*I linearized vector CIp30 was also transformed into the BWP17 wild-type strain and HSP12KO4 strain to generate the uridine and arginine prototrophic wild-type control strain BWT and HSP12KO5. The integration of *HSP12*-CIp30 at the *RP10* locus was confirmed by PCR using the primers RPS1-G1 (5’-GTGACGGACGTGTGGTTGTTAAGTC-3’) and H2 (5’- CAACGAAATGGCC TCCCCTACCACAG-3’). The integration was also confirmed by Southern blot analysis on *Spe*I digested genomic DNA with a 912 bp *RP10* probe, which was located 1285 bp upstream of *RP10* start codon. The restoration of *CaHSP12* expression was confirmed by qRT-PCR

To construct the *CaHSP12* overexpressing strain HSP12OE, the 507 bp Ca*HSP12* ORF and 204 bp downstream region were PCR amplified using primers HSP12F-PstI (5’-CCCCCCCTGCAGATGGCAACCTTTGCCCCCCTCTC-3’) and HSP12R- *BamH*I (5’- CCCCCCGGATCCGATCACTGGTCTGTCAACTGTCTCC-3’) (*Pst*I and *BamH*I sites are underlined) and cloned downstream of the strong *TEF2* promoter in pFM2 to generate pFM2-Ca*HSP12*. pFM2-Ca*HSP12* was lineralized at the *Hpa*I restriction site and transformed into the CAI4 strain. qRT-PCR and Western blot analysis were performed to confirm that *TEF2* promoter is constitutively expressing Ca*HSP12* at a high level.

*C. glabrata* is a haploid organism and possess only one Cg*HSP12* copy. Thus only one allele was needed to be deleted to generate the Cg*hsp12* null mutant. To delete Cg*HSP12*, the disruption cassette containing the *TRP1* gene which was flanked by the 532 bp downstream and the 525 bp upstream region of Cg*HSP12* was generated. The *TRP1* gene was amplified from the genomic DNA of *C. glabrata* Cg2001 strain by PCR using the primers TRP1F (5’-CGG TGC TCT ACA GGA ATC CG-3’) and TRP1R (5’-GGA AAT TAT AGC ATG AAT TCA TAG C-3’). The PCR fragment of the *TRP1* gene was then clone into pCR2.1-TOPO vector (Invitrogen) to obtain the Topo-TRP1 plasmid. The downstream region of *CgHSP12* were PCR amplified from the genomic DNA of *C. glabrata* Cg2001 strain using the primers CgHSP12KO5F (5’- CCC CCC GAG CTC TTA GAA CCA TTA GGT ATA GTG CCT C-3’) and CgHSP12KO5R (5’-CCC CCC GGA TCC TGT TAA ATT AAT ATG TGT GTG TTG TTG-3’) (*Sac*I and *Bam*HI sites are underlined), while the upstream region was amplified using the primers *Cg*HSP12KO3F (5’-CCC CCC GCG GCC GCA CTT GAA TAA CAT CTG ATG TTC AG-3’) and *Cg*HSP12KO3R 5’-CCC CCC CTC GAG GGG ATA TGA GAC AAG TTT CTT G-3’) (*Not*I and *Xho*I sites are underlined). The downstream and the upstream regions were cloned respectively into the *Sac*I-*Bam*HI and *Not*I-*Xho*I sites of the Topo-TRP1 plasmid. The disruption cassette was transformed into *Cg*2001TU to generate the *Cg*12KO Ura- strain (Cg12KOU). The transformants were selected on YNB minimal medium plates supplemented with 0.005% uridine. The correct integration was confirmed by PCR using the primers CgHSP12G1 (5’-GGA CAT GGT GTG CTG GGA AAC-3’) and CgHSP12T2 (5’- GGC GTC AAA GAC AAT GAC AAG -3’), CgHSP12T3 (5’- CCT GCT TGA AGC GCA GTT TC-3’) and CgHSP12G4 (5’-CAT ACC CAT GGG CTC TGT TAT AGG-3’). The absence of Cg*HSP12* expression was confirmed by qRT-PCR. To reintroduce Cg*HSP12* into the deletion strain, Cg*HSP12* was cloned into pEM13D. Cg12KOU was transformed by pEM13D and pEM13D-Cg*HSP12* to create Cg12KO and Cg12C respectively. The transformants were selected on YNB minimal medium plates without any supplement. The restoration of Cg*HSP12* expression was confirmed by using qRT-PCR.

**Analysis of two different alleles of *HSP12* using Southern blot**

Total 2 μg of genomic DNA, which was isolated from SC5314 and clinical isolates, were digested with 2 units of *Nde*I (New England Biolabs) and *Pac*I (New England Biolabs). Southern blot was carried out using probe which locates at 1110 bp upstream of the start codon of Ca*HSP12*. The 932 bp DNA template of probe were amplified from a genomic DNA of SC5314 wild-type by PCR using primers HSP12-SB-F (5’-CAT AGG GAA GAC TGA TAG CTG-3’) and HSP12-SB-R (5’-GCT GTG GTG CTG GGA ACA AG-3’).

**RNA extraction**

*C. albicans* strains were grown overnight in YNB minimal medium. Cells were diluted to OD_600_ 0.1 in 10 ml YNB minimal medium and grown to OD_600_0.5, ensuring growth to mid-log phase. To measure *C. glabrata* *HSP12* (Cg*HSP12*) mRNA levels under stress conditions, cells were grown from OD_600_ 0.1 to 0.5 before supplementing the media with the appropriate chemical for one additional hour (YNB acted as control). Total RNA was extracted after cell disruption using a bead beater Mikon-Dismembrator S (Sartorius, Goettingen, Germany) and purified using the RNeasy Mini Kit (Qiagen) according to the manufacturer’s instructions.

**Real-time RT-PCR**

qRT-PCR was carried out using iScript One-Step RT-PCR Kit with SYBR Green (Bio-Rad, Hertfordshire, UK) and the MiniOpticon system (Bio-rad). Primers for Ca*HSP12* (HSP12F 5’-AAA CAA TCC GAA CAA GCA GAG GGC-3’ and HSP12R 5’-TCC ACT CAC GTA TTC AGC AGC CTT-3’) and *ACT1* (F-ACT1 5’-CCT ACG TGT ACT TGT GCA AGG CAA-3’ and R-ACT1 5’-TAG TTG TGT GCA CTG AGC GTC GAA-3’) which was used as a control transcript. Primers for Cg*HSP12* (CgH12RTF 5’-AAC GCT GAA TCC TAC GCA GAC ACT-3’ and CgH12RTR 5’-TTC ACC ACC GTG AAC AGA CTT GGA-3’), and *CgACT1* (F-ACT 5’-TTA GAA CCA TTA GGT ATA GTG CCT C-3’ and R-ACT 5’- GGG ATA TGA GAC AAG TTT CTT G-3’) were used. The data were analyzed using Opticon Monitor version 3.1 analysis software. An average cycle threshold (Ct) value was obtained from the triplicates and normalized to the average Ct value of *ACT1*. The relative difference of expression was calculated in Delta Ct (2^–ΔCT^)

**3.2.4 5'-Rapid amplification of cDNA ends (RACE)**

5'-RACE reactions were performed with 1 µg total RNA extracted from *C. albicans* SC5314 wild-type strain, which had been grown in YNB minimal medium, using the FirstChoice RLM-RACE kit (Ambion, Foster City, USA). RNA processing, adaptor ligations and reverse transcription were implemented according to the manufacturer’s instructions. 5'-RLM-RACE-PCRs were analyzed with a 5’-RACE outer primer provided in the kit and RTR2 (5’-TTT CAG CAA GGG TCT TTG CTT CGC-3’). The PCR products were visualized on a 2 % agarose gel electrophoresis. The fragments of interest were gel extracted, purified and sent for sequencing (MWG).

**Expression and purification of CaHsp12p from *E. coli***

*Ca*Hsp12 protein was expressed as a glutathione S-transferase (GST) fusion protein and purified using Glutathione sepharose 4B column (GE healthcare). Ca*HSP12* orf DNA fragment was generated using PCR with primers HSP12GSTF (5’- CCC CCC GGA TCC GCA ACC TTT GCC CCC CTC TC-3’) and HSP12GSTR (5’- CCC CCC GAA TTC GAT CAC TGG TCT GTC AAC TGT CTC C-3’) in which HSP12GSTF contains *Bam*HI restriction site (underlined sequence) and HSP12GSTR contains *Eco*RI restriction site (underlined sequence). Ca*HSP12* was ligated to the GST gene of pGEX-6P-2 as *Bam*HI-*Eco*RI fragments and introduced into *E. coli* BL21 pLysS cells. Purification of the GST-*Ca*Hsp12p fusion protein and removal of GST tag by PreScission Protease cleavage were carried according to the manufacturer’s instructions.

**Protein expression in yeast and Western blotting**

For the study of the Hsp12p expression in different growth phase, overnight *C. albicans* cultures were diluted to OD_600_ of 0.1 in unbuffered YNB minimal medium and incubated at 37 °C. Samples were collected at various time points as indicated in figure 2B. Protein samples from *S. cerevisiae* were collected using the same method, but the cells were grown at 30 °C.

For the study of the Hsp12p expression under different stress conditions, overnight *C. albicans* cultures were diluted to OD_600_ of 0.1 in unbuffered YNB minimal medium and incubated at 37 °C. The cells were grown until OD_600_ of 0.5, ensuring growth was to the mid-exponential phase. The culture was divided into several aliquots; one sample was maintained as control and the other were treated with the chemicals which were added to the medium at the following the final concentrations: 0.1 or 0.3 M NaCl (Fisher scientific), 0.1 or 0.3 M sorbitol (Sigma-Aldrich), 0.4 or 1.0 mM hydrogen peroxide (Sigma-Aldrich), 0.1 mM or 0.5 mM CdSO_4_ (Fisher scientific), 100 μM farnesol (Sigma-Aldrich), 100 μM dodecanol (Sigma-Aldrich), 4 μg ml^-1^ fluconazole (Sigma-Aldrich), 4 μg ml^-1^ ketoconazole (Sigma-Aldrich), 4 μg ml^-1^ itraconazole (Sigma-Aldrich) or 2.5 μg ml^-1^ amphotericin B (Sigma-Aldrich). The cells were then grown for a further hour. For the heat shock experiment, cells were grown at 30 °C or 37 °C and had a temperature shift to 37 °C or 45 °C for 10 min. Heat shock treated cells were then recovered by growing them at the initial temperature for 1 h. Protein samples from *S. cerevisiae* were collected using the same method, but the cells were cultured at 30 °C.

For the investigation of Hsp12p in response to various pH or to CO_2_, cells were grown in YNB minimal medium at pH 4 or 7.5 in air or 5.5 % CO_2_ until the mid-log phase. The pH of the medium was tested following pre-equilibration in a test flask and found to remain constant to within 0.5 pH units of the set value.

Cell samples were pelleted by centrifugation at 13,000 *× g* 4 °C for 5 min and resuspended in 200 μl lysis buffer (2 % Triton X-100, 1 % SDS, 100 mM NaCl, 10 mM Tris pH 8.0 and 1 mM EDTA) with 0.1 × protease inhibitor cocktail tablets (Roche, Lewes, UK). Small drops of culture were pipetted in liquid nitrogen Frozen yeast drops were shaken severely with glass bead using a bead beater Mikon-Dismembrator S at 2,300 rpm for 2 min. Total proteins extracts were stored at -20°C.

Whole cell lysates were assessed by Western Blotting using an anti-*Ca*Hsp12p primary antibody (1:125 dilution in 5 % fat free milk) or an anti-*Sc*Hsp12p primary antibody, generated by Praekelt and Meacock (1:1000 dilution in 5 % fat free milk), and made available by Mick Tuite (University of Kent). Blots were then hybridized by goat anti-rabbit IgG (whole molecule)-horseradish peroxidase secondary antibody (1:1000 dilution in 5 % fat free milk; Sigma-Aldrich). Equal protein loading was determined by probing the blot with anti-actin antibody (1: 1000 dilution; Sigma-Aldrich).

Subsequent data analysis was performed using NIH Image J software. The densitometry units for the Hsp12p were normalized to the densitometry units for actin. These normalized densitometry units were then expressed relatively to control (unstressed condition or wild-type strain) as a relative densitometry unit (RDU).

**Growth rate determination**

Strains from overnight culture were subcultured in 50 ml YNB minimal medium to achieve an OD_600_ of 0.1 and then grown at 37 °C in a shaking incubator. The OD_600_ was measured every hour using a spectrophotometer (Eppendorf). The growth curve was plotted by OD_600_ against time. Triplicate biological experiments have been performed.

**Cell aggregation assay**

The aggregation assay was modified from the one described by Eboigbodin and Biggs . The assay was performed on cells grown in YNB liquid medium after 4 h. 1 ml of culture was transferred into the steady cuvettes. Since the cell aggregates settled to the bottom of cuvettes, OD_600_ corresponding to cells at the upper part of the cuvettes was measured at the time points indicated. The percentage of cells which sedimented to the bottom of the cuvettes was calculated according to following equation:

% of cell sedimented = OD_I_ – OD_t_ / OD_I_ x 100; where OD_I_ is the initial OD taken at time 0 and OD_t_ is the OD taken at the time points indicated. Triplicate biological experiments have been performed.

**Microtitre plate cell adhesion XTT reduction assay**

Biofilms from different strains were produced on sterilized, polystyrene, flat-bottom 96-well microtitre plates (Sigma-Aldrich). The OD_600_ of the overnight cultures was adjusted to 1.0. 100 μl of cell suspension was then transferred into each well (in triplicates) of a microtitre plate and incubated at 37 °C for 24 h to allow the yeasts to adhere and form a biofilm on the surfaces of the wells. 100 μl of liquid medium without cell suspension was also added into wells in three replicas as controls. Biofilm formation of the strains were quantified by the 2,3-bis(2-methoxy-4-nitro-5-sulfophenyl)-2H-tetrazolium-5-carboxanilide (XTT) reduction assay .

**Filamentation assay**

To assay *C. albicans* hyphal growth, strains from overnight cultures were transferred to 20 ml liquid YNB minimal medium at pH 4 or pH 7 or supplemented with 5 % serum with or without 100 μM farnesol to achieve the OD_600_ of 0.1. Culture was grown at 37 °C in air or in 5.5 % CO_2_ in a shaking incubator. Cells were then examined and the germ tube formation was counted every 30 min under a light microscope (LEITZ DMRB). Triplicate biological experiments have been performed.

**Antifungal drug and stress sensitivity tests**

The overnight cultures were diluted in liquid YNB minimal medium to an OD_600_ of 2 and further diluted 10-, 100-, 1000-, 10000- fold. The dilutions (5 μl) were spotted onto YNB plates supplemented with either **0.1 – 1 M** sodium chloride, **0.1 – 1 M** sorbitol, 0.5 – 50 mM H_2_O_2_, **0.05 - 0.7 mM** menadione, 0.1 mM – 0.5 mM CdSO_4_, 0.1 – 0.6 mM Congo red, 25– 40 μg ml^-1^ calcofluor white, 2 – 50 mM caffeine, 0.01 – 0.07 % SDS, 4 μg ml^-1^ itraconazole, ketoconazole and fluconazole, 2.5 μg ml^-1^ amphotericin B and 5 nM rapamycin. Plates were incubated for 24 h at 37 °C. Replicate biological experiments have been performed.

**Virulence test in *Drosophila* model**

The Toll transheterozygotes flies were obtained by crossing the flies with a loss of the function allele of Toll (*Tl^1-RXA^*; obtained from the Tübingen *Drosophila* Stock Collection) and the flies with a thermosensitive allele of Toll (*Tl3*; obtained from the Bloomington Stock Centre). All the stocks were kept on standard fly medium in vials at 25 °C, except during infection experiments where the flies were incubated at 30 °C.

The infection of 2-to-5 day old adult flies with the *C. albicans* strains was carried out by using a thin sterile needle dipped in a *C. albicans* suspension (~ 10^10^ cell ml^-1^) and injecting into the thorax. Following infection, mortality was monitored for total 40 h. The infection experiments were performed three independent times, with 15 flies per experimental group.

**References**

1. Fonzi WA, Irwin MY (1993) Isogenic strain construction and gene mapping in *Candida albicans*. Genetics 134: 717-728.

2. Wilson RB, Davis D, Mitchell AP (1999) Rapid hypothesis testing with *Candida albicans* through gene disruption with short homology regions. J Bacteriol 181: 1868-1874.

3. Wendland J (2003) PCR-based methods facilitate targeted gene manipulations and cloning procedures. Curr Genet 44: 115-123.

4. Gietz RD, Woods RA (2002) Transformation of yeast by lithium acetate/single-stranded carrier DNA/polyethylene glycol method. Methods Enzymol 350: 87-96.

5. Dennison PM, Ramsdale M, Manson CL, Brown AJ (2005) Gene disruption in *Candida albicans* using a synthetic, codon-optimised Cre-loxP system. Fungal Genet Biol 42: 737-748.

6. Praekelt UM, Meacock PA (1990) *HSP12*, a new small heat shock gene of *Saccharomyces cerevisiae*: analysis of structure, regulation and function. Mol Gen Genet 223: 97-106.

7. Eboigbodin KE, Biggs CA (2008) Characterization of the extracellular polymeric substances produced by *Escherichia coli* using infrared spectroscopic, proteomic, and aggregation studies. Biomacromolecules 9: 686-695.

8. Jin Y, Yip HK, Samaranayake YH, Yau JY, Samaranayake LP (2003) Biofilm-forming ability of *Candida albicans* is unlikely to contribute to high levels of oral yeast carriage in cases of human immunodeficiency virus infection. J Clin Microbiol 41: 2961-2967.

9. Hiller E, Heine S, Brunner H, Rupp S (2007) *Candida albican*s Sun41p, a putative glycosidase, is involved in morphogenesis, cell wall biogenesis, and biofilm formation. Eukaryot Cell 6: 2056-2065.
